# Cembrene Diterpenoids with Ether Linkages from *Sarcophyton ehrenbergi*: An Anti-Proliferation and Molecular-Docking Assessment

**DOI:** 10.3390/md15060192

**Published:** 2017-06-21

**Authors:** Mohamed-Elamir F. Hegazy, Abdelsamed I. Elshamy, Tarik A. Mohamed, Ahmed R. Hamed, Mahmoud A. A. Ibrahim, Shinji Ohta, Paul W. Paré

**Affiliations:** 1 Phytochemistry Department, National Research Centre, 33 El-Bohouth St., Dokki, Giza 12622, Egypt; elamir77@live.com (M.-E.F.H.); tarik.nrc83@yahoo.com (T.A.M.); n1ragab2004@yahoo.com (A.R.H.); 2 Natural Compounds Chemistry Department, National Research Centre, 33 El-Bohouth St., Dokki, Giza 12622, Egypt; elshamynrc@yahoo.com; 3 Biology Unit, Central Laboratory for Pharmaceutical and Drug Industries Research Division, National Research Centre, 33 El-Bohouth St., Dokki, Giza 12622, Egypt; 4 Computational Chemistry Laboratory, Chemistry Department, Faculty of Science, Minia University, Minia 61519, Egypt; m.ibrahim@compchem.net; 5 Graduate School of Biosphere Science, Hiroshima University, 1-7-1 Kagamiyama, Higashi-Hiroshima 739-8521, Japan; ohta@hiroshima-u.ac.jp; 6 Department of Chemistry and Biochemistry, Texas Tech University, Lubbock, TX 79409, USA

**Keywords:** *Sarcophyton ehrenbergi*, soft coral, terpenes, cembranoids, cytotoxic activity, molecular docking

## Abstract

Three new cembrene diterpenoids, sarcoehrenbergilid A–C (**1**–**3**), along with four known diterpenoids, sarcophine (**4**), (+)-7α,8β-dihydroxydeepoxysarcophine (**5**), sinulolide A (**6**), and sinulolide B (**7**), and one steroid, sardisterol (**8**), were isolated and characterized from a solvent extract of the Red Sea soft coral *Sarcophyton ehrenbergi*. Chemical structures were elucidated by NMR and MS analyses with absolute stereochemistry determined by X-ray analysis. Since these isolated cembrene diterpenes contained 10 or more carbons in a large flexible ring, conformer stabilities were examined based on density functional theory calculations. Anti-proliferative activities for **1**–**8** were evaluated against three human tumor cell lines of different origins including the: lung (A549), colon (Caco-2), and liver (HepG2). Sardisterol (**8**) was the most potent of the metabolites isolated with an IC_50_ of 27.3 µM against the A549 cell line. Since an elevated human-cancer occurrence is associated with an aberrant receptor function for the epidermal growth factor receptor (EGFR), molecular docking studies were used to examine preferential metabolite interactions/binding and probe the mode-of-action for metabolite-anti tumor activity.

## 1. Introduction

Cembrane diterpenoids are a large and structurally diverse group of natural products isolated from both terrestrial and marine organisms [1]. The 14-membered ring structure is biosynthetically formed from the cyclization of the geranylgeraniol precursor between carbons 1 and 14. The cembranoid diterpene, sarcophytol A, first isolated from the Okinawan soft coral *Sarcophyton glaucum* and found to exhibit strong inhibitory activity against tumor promoters [2], led to the subsequent isolation of hundreds of cytotoxic cembranoids from plant and marine sources [3]. *Sarcophyton* soft coral species are characterized by the production of cembrene-type diterpenoids [4,5,6,7,8,9,10] and these cyclic diterpenes usually exhibit cyclic ether, lactone, or furane moieties around the cembrane framework [11,12]. From a biomedical perspective, cembranoid diterpenes exhibit a diverse range of biological protection against tumors, inflammation, and fish toxins (ichthyotoxic), as well as microbial and/or viral infections [4,5,13]. With cancer occurrence and mortality associated with cancer increasing in the U.S. and around the world, the exploration of cytotoxic agents for improved cancer treatment via chemotherapy is a global priority. In fact, the World Health Organization (WHO) estimates that malignant neoplasms are ranked as the second leading cause of death globally. In 2012, 14.1 million newly-diagnosed cancer cases were reported, with 8.2 million deaths directly associated with cancer; these incidence and mortality numbers are estimated to increase by ca. 150% by 2030 [14,15].

In a continuing effort to characterize soft coral metabolites from the Red Sea with biological activity [6,7,8], herein is reported three new cembrene diterpenoids, as well as known diterpenoids and a polyoxygenated steroid isolated from *Sarcophyton ehrenbergi*. In this study, the anti-proliferative potential of the isolated compounds against three human tumor cell lines were evaluated. To probe the mode-of-action, molecular docking studies were performed with the epidermal growth factor receptor (EGFR), a large family of transmembrane receptors that normally regulate key events associated with cell growth, differentiation, and migration. An aberrant receptor function has been linked to elevated cancer occurrence. 

## 2. Results and Discussion

### 2.1. Identification and Structure Elucidation

**Figure 1:**
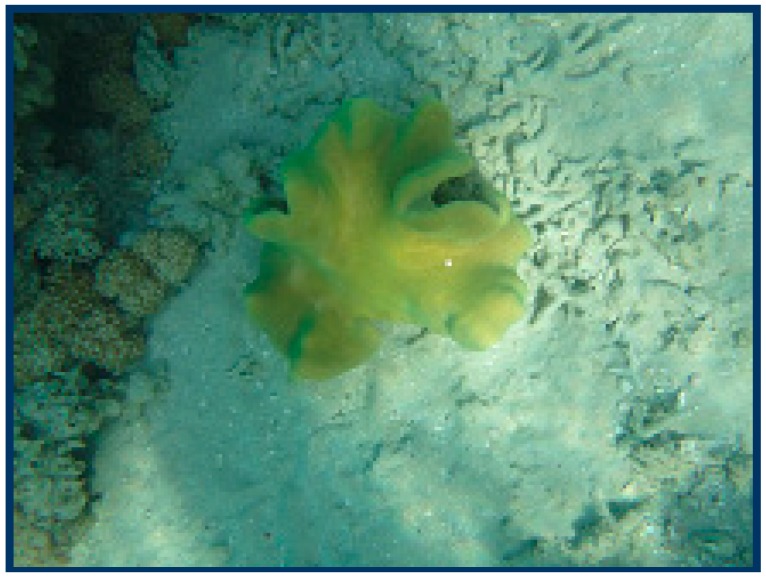
Soft coral *Sarcophyton ehrenbergi* photographed in its native Red Sea habitat; the width of the species shown is ca. 20 cm.

As part of the continuing investigation for biologically active constituents from Egyptian Red Sea costal soft corals [6,7,8,16], reported here is the chromatographic fractionation and purification of a methylene chloride:methanol (1:1) extract from *S. ehrenbergi* (Figure 1).

**Figure 2:**
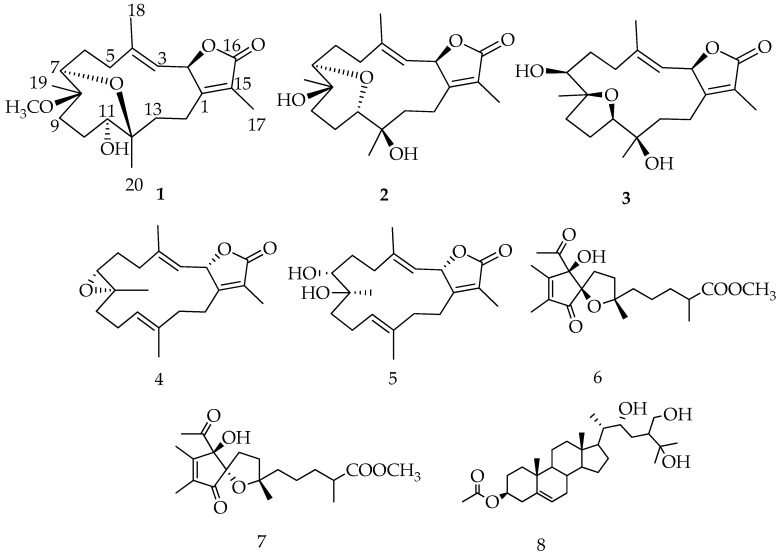
Structures of metabolites **1**–**8**.

Compound **1** was obtained as white crystals with an optical rotation of ${\left[\alpha \right]}_{25}^{D}$ −6.9 in CHCl_3_. HRESIFTMS analysis showed a molecular ion peak at *m*/*z* 387.2142 [M + Na]^+^ (calcd. 364.2250), corresponding to the molecular formula of C_21_H_32_O_5_. The IR spectrum showed characteristic bands at 3450 cm^−1^ (OH) and 1754 cm^−1^ (CO). The ^1^H NMR spectrum (Table 1) exhibited three oxygenated protons at δ_H_ 5.50 (d; *J* = 10.10 Hz); δ_H_ 3.24 (d; *J* = 6.90 Hz); and δ_H_ 3.37 (m). Only one olefinic proton at δ_H_ 4.94 d; *J* = 10.10 Hz was attributed to a tri-substituted double bond; four signals at δ_H_ 1.76 s, 1.86 s, 1.05 s, and 0.98 s were identified as methyls, in addition to one methyl of a methoxy group at δ_H_ 3.13 s. Twenty one carbon signals were observed in the ^13^C NMR spectrum and classified by DEPT analysis as five methyls (including one methyl of the methoxy group at δ_C_ 49.0), six methylenes, four methines, and six quaternary carbons (including the carbonyl group of the lactone ring δ_C_ 175.9 (Table 1)). The spectrum also revealed the presence of four olefinic carbon signals at δ_C_ 122.3, 164.3, 119.4, and 141.6; three oxymethine carbons at δ_C_ 81.0, 78.5, and 79.0; and one oxygenated quaternary carbon of an epoxy at δ_C_ 78.3. The most oxygenated down-field carbon signal indicated the presence of an ether linkage that was functionality confirmed by HRESIFTMS. These spectroscopic data were consistent with a cembrene diterpenoid based on spectroscopic data reported for other *Sarcophyton* species [4,13] (Figure 2). Six degrees of unsaturation were deduced, suggesting a tricyclic skeleton. The correlation of the oxygenated proton at δ_H_ 5.50 (d; *J* = 10.10 Hz) with the olefinic signal at δ_H_ 4.94 (d, *J* = 10.10 Hz) in DQF-COSY, as well as with quaternary olefinic carbons at δ_C_ 141.6 and δ_C_ 164.3, allowed the assignments of H-2, H-3, C-4, and C-1 of a cembrene diterpenoid, respectively [9,10].

**Figure 3:**
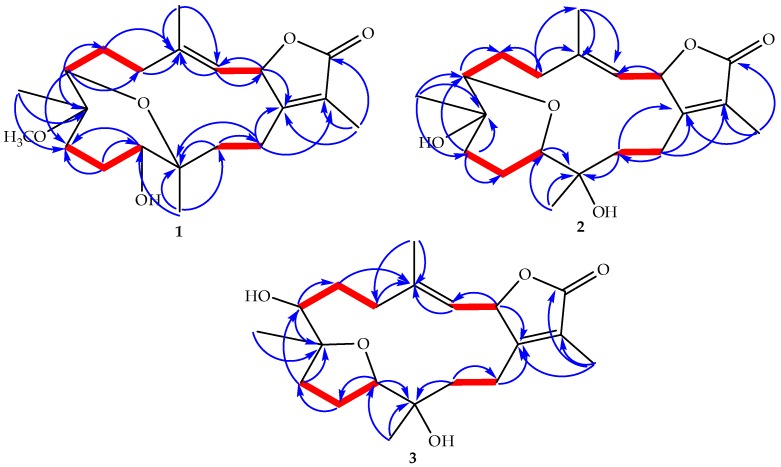
Selected ^1^H-^1^H COSY (▬) and HMBC (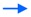) correlations of **1**–**3**.

**Figure 4:**
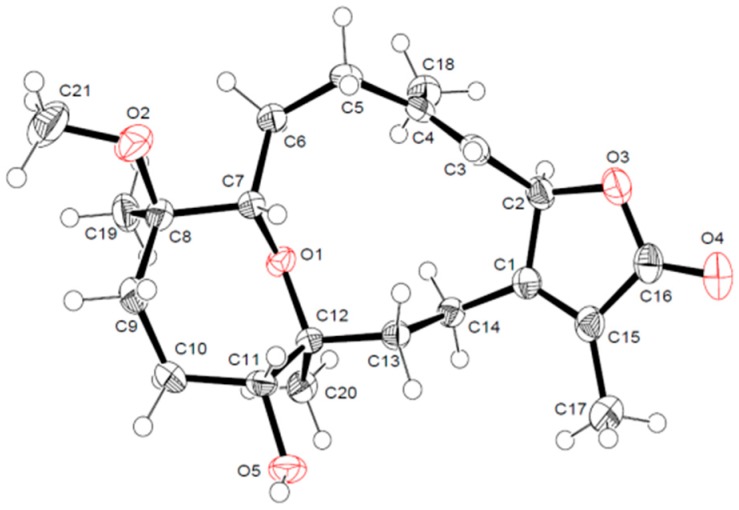
ORTEP depictions of cembrenoid **1** with oxygens (O1–O5) labeled in red.

The HMBC correlation of a methyl signal at δ_H_ 1.76 (s) with C-1 and a keto group at δ_C_ 175.9 allowed for the assignment of H-17 and C-16, respectively, and indicated the location of a lactone ring, including C-1/C-2. The observed HMBC correlation between H-3 and an olefinic methyl signal at δ_C_ 17.0 and a methylene signal at δ_C_ 40.9 allowed for the assignment of H-18 (δ_H_ 1.86, s) and H-5 [δ_H_ 2.07, t (*J* = 13.08)], respectively, which was confirmed by HMQC analysis. A doublet oxygenated methine signal at δ_H_ 3.24 (*J* = 6.90) correlated with a methyelene multiplet at δ_H_ 2.44/2.14 in DQF-COSY and C-5 in HMBC allowed for the assignment of H-7 and H_2_-6, respectively. Additionally, HMBC correlations of the methyl singlet at δ_H_ 1.05 with H-7 and an oxygenated quaternary carbon atom at δ_C_ 80.0, as well as the methyelene signal at δ_C_ 36.4, allowed for the assignment of H_3_-19 (δ_C_ 17.1), C-8, and C-9, respectively. The oxygenated signal at δ_H_ 3.37 (m) was assigned to H-11 (δ_C_ 79.0) based on an HMBC correlation with C-9 and a methyl signal at δ_C_ 17.6 (C-20). Correlations were observed between δ_H_ 1.45 (m, H-13)/δ_H_ 1.95 (m, H-14) and C-20 in DQF-COSY and HMBC analyses, respectively (Figure 3). The location of a characteristic methoxy group signal at δ_H_ 3.13 (δ_C_ 49.0) was confirmed to be at C-8 via an HMBC correlation. The complete assignment of **1**, as well as the ether linkage between C-7/C-12 and the presence of the hydroxyl group at C-11, were established by NMR and HRESIFTMS data; structural confirmation including absolute configuration was established unambiguously using the anomalous scattering of Cu Kα radiation with the Flack parameter [17,18] being refined to 0.09 (3) (Figure 4).

**Figure 5:**
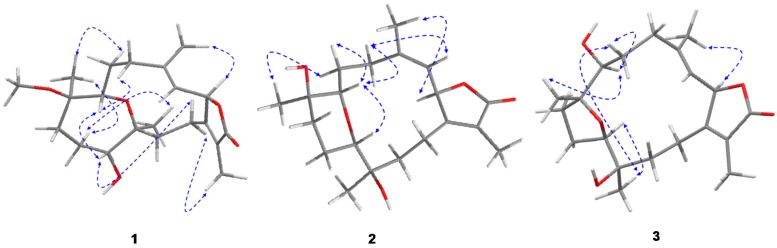
NOESY correlations for **1**–**3**.

The γ-lactone- (H-2) and olefinic-proton (H-3) vicinal coupling (10.10 Hz) established a *cis* configuration [8]. The four methyl groups exhibited NOSEY correlations with alpha protons consistent with the X-ray assignment of all methyl groups below the ring (e.g., CH_3_-17 with H-14a, CH_3_-18 with H-2, CH_3_-19 with H-6a/H-10a, and CH_3_-20 with H-10a) and absolute stereochemistry of 8*R* and 12*S* (Figure 5). NOSEY correlations between H-7 and H-5b, as well as H-11 and H-14b, were also consistent with 7*R* and 11*R* configurations. From this consistent x-ray and NMR data, **1** was assigned as 2*S*,16:7*R*,12*S*-diepoxy-11*α*-hydroxy-8*β*-methoxy-16-keto-cembra-1*E*,3*E*-diene (sarcoehrenbergilid A).

Compound **2** was obtained as a white powder with an optical rotation of ${\left[\alpha \right]}_{25}^{D}$ −3.7 (*c* 0.0027, CHCl_3_). HRESIFTMS analysis showed a molecular ion peak at *m*/*z* 373.1986 [(M + Na)^+^] (calcd. 350.2093), implying six degrees of unsaturation. The IR spectrum exhibited characteristic bands at 3447 cm^−1^ (OH) and 1747 cm^−1^ (CO). ^13^C NMR and DEPT spectral data (Table 1) showed 20 carbon resonances that distributed in the configuration of four methyls, six methylenes, four methines, and six quaternary carbons. Chemical shift data indicated the same cembrenoid backbone, containing diagnostic carbon signals associated with the lactone ring including a carbonyl signal C-16 (δ_C_ 175.6), three olefinic carbons at C-15, C-1, C-3, and C-4 (δ*_C_* 122.5, 164.4, 117.8, and 146.0, respectively), and C-2 (δ_C_ 79.6). The spectra data closely matched a cemberene compound reported by Sawant et al. in 2004 [19], except for a large down field carbon signal difference at C-12 compared with the previously published structure tertiary carbon, which had a methyl substitution (δ_C_ 38.0). Compound **2** was proposed to contain a hydroxylated quaternary carbon at C-12 which would explain the downfield shift to δ*_C_*72.8 and a 16 AMU addition compared to the previously published compound [20]. The addition of a hydroxyl group at C-12 was consistent with a H_3_-20 downfield shift from δ_H_ 0.88 to δ_H_ 1.16 and a C-20 downfield shift from δ_C_ 17.2 to δ_C_ 23.8 without versus with a C-12 hydroxyl group. HMBC correlations of H_3_-20 (δ_H_ 1.16 s) with C-12 (δ_C_ 72.8), C-11 (δ_C_ 78.5), and C-13 (δ_C_ 35.0) were also consistent with the hydroxylation of C-12 (Figure 3). 

Similar to **1**, the γ-lactone- (H-2) and olefinic-proton (H-3) vicinal coupling (10 Hz) established a *cis* configuration [8]. Also similar to **1**, the NOSEY data for **2** showed a correlation between H-6b and H-7, indicating that the epoxy ring at C-7 is below the ring while H-6a correlates with CH_3_-19, establishing that the relative stereochemistry for the methyl is above the ring (Figure 5). H-7, which is assumed to be in the beta position from the previous NOSEY correlation, also correlates with H-11, indicating that the other epoxide ring attachment is in an alpha configuration. Finally, a NOSEY correlation between H-10a and CH_3_-20 indicates that the methyl group is in an alpha orientation. Thus, **2** was confirmed to be 2*S*,16: 7*R*,11*R*-diepoxy-8β,12β-dihydroxy-16-keto-cembra-1*E*,3*E*-diene (sarcoehrenbergilid B).

Compound **3** was obtained as a white powder with a negative optical rotation of ${\left[\alpha \right]}_{25}^{D}$ −6.6. HRESIFTMS analysis exhibited a molecular ion peak at *m*/*z* 373.1985 [(M + Na)^+^] (calcd. 350.2093), corresponding to the molecular formula C_20_H_30_O_5_ with six degrees of unsaturation. The IR spectrum showed characteristic bands at 3445 cm^−1^ (OH) and 1747 cm^−1^ (CO). Twenty carbon resonances were exhibited in the ^13^C NMR and DEPT spectrum (Table 1); four methyls, six methylenes, five methines, and five quaternary carbons. The spectroscopic data of **3** are similar to a previously isolated diterpenoid from *S. trocheliophorum*, trocheliophorol [21], except for the presence of a hydroxyl unit at C-12 (δ_C_ 70.1) instead of an exomethylene. The location of the C-12 hydroxyl group was confirmed by HMBC correlations with a methyl singlet H_3_-20 (δ_H_ 1.13 s); correlations were also observed between H_3_-20 and δ_C_ 85.1 (C-11) and δ_C_ 31.2 (C-13) (Figure 3). 

Similar to compounds **1** and **2**, a γ-lactone- (H-2) and olefinic-proton (H-3) vicinal coupling (10 Hz) established a *cis* configuration [8]. A NOESY correlation between H-6a with H-7 indicated that the C-7 hydroxyl group is in a beta orientation and a H-6a correlation with CH_3_-19 indicated that the methyl at C-8 is in an alpha configuration (Figure 5). A NOSEY correlation was also observed between H-6a and CH_3_-20, indicating that the hydroxyl at C-12 is in the beta orientation. Finally, a NOSEY correlation between CH_3_-20 and H-11 indicated that the epoxide connection at C-11 is the same as C-8, both in alpha configurations. From the above spectral data, **3** was established as 2*S*,16: 8*S*,11*S*-diepoxy-7*β*,12*α*-dihydroxy-16-keto-cembra-1*E*,3*E*-diene (sarcoehrenbergilid C).

**Figure 6:**
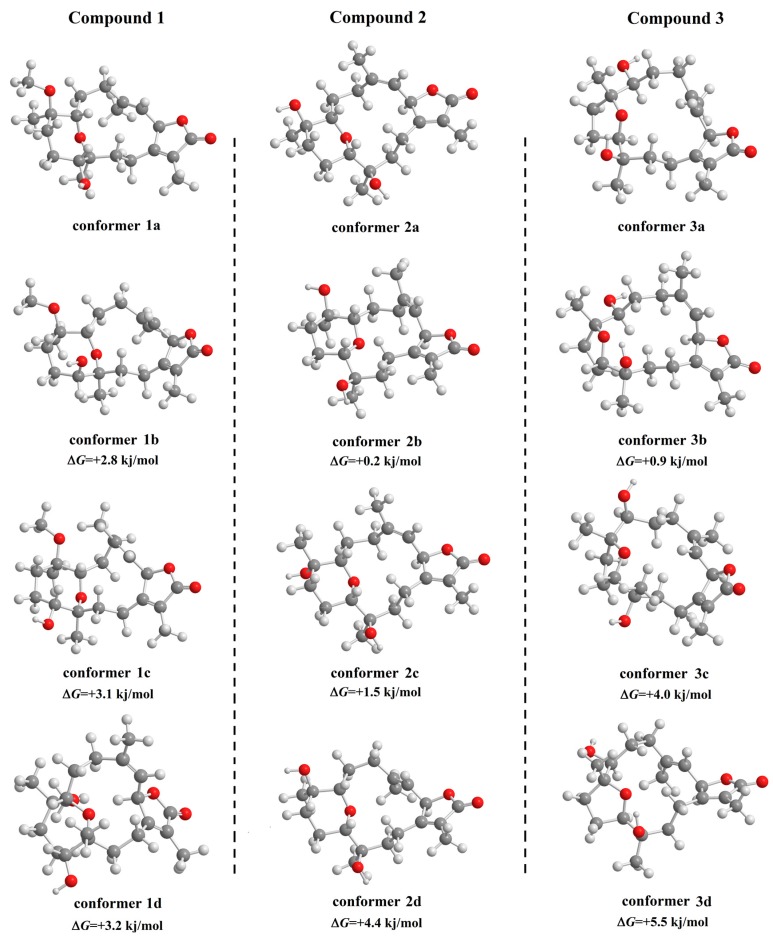
Optimized confirmers for **1**–**3,** as well as the next three higher free-energy conformers.

With the large flexible ring systems for compounds **1**–**3,** many conformers are possible. To predict the most stable form, molecular modeling calculations were performed to estimate the lowest energy state. Conformation ensembles were generated using a MMFF94 molecular mechanics force field within a 10 kJ/mol window, providing 101, 98, and 106 conforms for **1**, **2**, and **3**, respectively. Each generated conformer was subjected to energy-minimization at a B3LYP/6-31G* level of theory and thereafter, the corresponding free energy was calculated on the optimized structure. The Boltzmann population was estimated based on the calculated relative free energies for each conformer with respect to the lowest free energy conformer at 298 K (Table S1). The lowest and next three higher free energy conformers for **1**–**3** are shown (Figure 6). All conformations with populations higher than 1% are shown in Figures S22–S24. The optical rotation of all conformations was theoretically calculated and the results are summarized in Table S1. According to the calculated free energies and optical rotation, the lowest compound conform free energy is in strong agreement with the elucidated structures (Figure 6). For **2**, the structure is more stable than a previously reported conformer (Figure S23, **2f**) by −7.4 kJ/mol.

In addition to the three new metabolites (**1**–**3**), four known compounds, sarcophine (**4**), (+)-7α,8β-dihydroxydeepoxysarcophine (**5**) [6], sinulolide A (**6**), and B (**7**) [22], and one steroid, sardisterol (**8**) [23], were identified from the coral extract (Figure 2).

### 2.2. Anti-Proliferative Activity against Cancer Lines

Isolated compounds **1**–**8** were evaluated for their anti-proliferative activity against three human tumor cell lines originating from the lung (A549), colon (Caco-2), and liver (HepG2) tissue based on an MTT reduction assay. The treatment of a human lung tumor cell line revealed differential anti-proliferative effects (Table 2). Among all tested compounds, sardisterol (**8**) had the most potent effect on A549 cells with a concentration-dependent loss of cell proliferation compared to a DMSO solvent control. Sardisterol was isolated for the first time from the marine soft coral *S. digitatum* [23]. To the best of our knowledge, this is the first report of anti-proliferative activity for **8** against tumor cells (Table 2 and Figure S25). The treatment of HepG2 cells with increasing concentrations of **1**–**8** revealed differential anti-proliferative potential (Table 2 and Figure S27). Compounds **3** and **8** showed a moderate inhibition (IC_50_ = 53.8 and 56.8 µM, respectively). Compounds **1**, **3**, **5**, **6**, and **7** exhibited IC_50_ values between 63.1 and 98.6 µM.

### 2.3. Molecular Docking Studies

**Figure 7:**
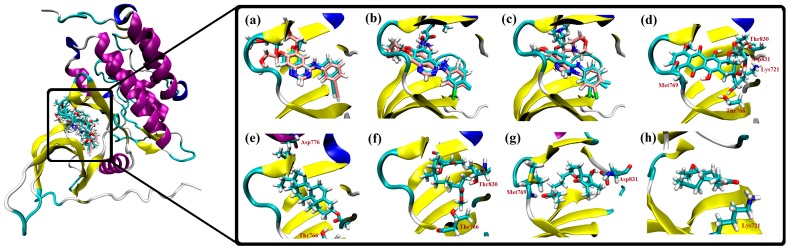
Crystal structure (in cyan) and predicted docking pose (in pink) of (**a**) erlotinib; (**b**) afatinib; and (**c**) gefitinib; and AMBER-based minimized docked structures of (**d**) doxorubicin; (**e**) **8**; (**f**) **7**; (**g**) **6**; and (**h**) **4** with EGFR kinase domain (PDB code: 1M17).

The epidermal growth factor receptor (EGFR) is a tyrosine kinase receptor that is overexpressed in many tumor cell types and reported as a cause for some non-small-cell lung carcinomas [24]. Based on the hypothesis that anti-proliferation activity with the A549 cell line is associated with EGFR inhibition [25], receptor binding to an ATP binding site domain of EGFR kinase by isolated metabolites was examined. Doxorubicin, a known effector molecule that binds to the ATP binding domain of EGFR, was initially used as a positive control. The ability of **8** (the most active metabolite) and **4** (the least active) were examined using molecular docking simulations. Simulations were conducted with AutoDock 4.2 software with an initial assessment of docking done by comparing the docked pose of the co-crystallized inhibitor ligand erlotinib with the experimental pose (PDB code: 1M17). To further provide a predictive picture as to where the compounds are positioned in terms of direct interactions with EGFR, docking scores for the newly isolated natural products are provided and two other EGFR inhibitors, afatinib and gefitinib, as well as the positive control doxorubicin (Table S2). Per the predicted binding poses, AutoDock accurately reproduced the crystal structure observed binding modes of erlotinib, afatinib, and gefitinib (Figure 7a–c). To reveal the binding features and possible interactions, compounds were subjected to molecular docking simulation followed by AMBER-based molecular mechanical minimization and MM/GBSA binding energy calculations. The calculated binding energies are in good agreement with experimental data, with a correlation coefficient *R*^2^ of 0.96 (Table S2, Figure S28). Per the minimized doxorubicin complex (Figure 7d), the highest potency of doxorubicin (ΔGMM/GBSA = −54.72 kcal/mol) may be attributed to its ability to form five hydrogen bonds with Lys_721_, Thr_766_, Met_769_, Thr_830_, and Asp_831_, with bond lengths of 1.92, 1.76, 1.82, 1.92, and 1.94 Å, respectively. For **6**–**8**, ligand-receptor interactions are stabilized by two hydrogen bonds with the active site (Figure 7e–g), resulting in a stronger binding energy value of −41.18 kcal/mol in the case of **8**. For **4**, the lower binding energy may be due to the formation of only one hydrogen bond with the active site Lys_721_ (Figure 7h). Several van der Waals and hydrophobic interactions were observed between the added marine ligand and amino acids in the active site including Leu_865_, Leu_694_, and Val_702_. To assess the stability of the predicted ligand-receptor complex, a short molecular dynamics simulation of 2.5 ns was performed for **8** in the complex with the EGFR receptor. The corresponding hydrogen bond distance between **8** and a carboxylate oxygen atom of Asp_776_ was then measured and averaged over the simulation time (Figure S29). According to the calculation, **8** can be considered stable, with an average hydrogen bond length of 2.16 Å with Asp_776_.

## 3. Experimental Section

### 3.1. General Experimental Procedures

Specific rotation was measured with a JASCO P-2200 polarimeter (JASCO Corporation, Tokyo, Japan) and the IR spectra were collected on a JASCO FT/IR-6300 spectrometer (JASCO Corporation, Tokyo, Japan). HR-ESI-FT-MS was carried out using a Thermo Fisher Scientific LTQ Orbitrap XL mass spectrometer (Waltham, MA, USA) at the Natural Science Center for Basic Research and Development (N-BARD), Hiroshima University. The ^1^H (600 MHz) and ^13^C (150 MHz) NMR spectra were recorded on a JEOL JNM-ECA 600 spectrometer (JEOL Ltd, Tokyo, Japan) with tetramethylsilane as an internal standard. Purification was run on a Shimadzu HPLC system equipped with a RID-10A refractive index detector and compound separation was performed on YMC-Pack ODS-A (YMC CO., LTD., Tokyo, Japan, 250 × 4.6 mm i.d., 5 µm) and (250 × 10 mm i.d., 5 µm) columns for analytical and preparative separation, respectively. Chromatography separation included normal-phase Silica gel 60 (230–400 mesh, Merck, Darmstadt, Germany), which was used for column chromatography. Pre-coated silica gel plates (Kieselgel 60 F_254_, 0.25 mm, Merck, Darmstadt, Germany) were used for TLC analyses. Spots were visualized by heating after spraying with 10% H_2_SO_4_.

### 3.2. Animal Material

Soft coral *Sarcophyton ehrenbergi* was collected from the Egyptian Red Sea off the coast of Hurghada in March 2015. The soft coral was identified by M Al-Hammady with a voucher specimen (03RS27) deposited in the National Institute of Oceanography and Fisheries, marine biological station, Hurghada, Egypt.

### 3.3. Extraction and Separation

Frozen soft coral (5.2 kg, total wet weight) was chopped into small pieces and extracted with methylene chloride/methanol (1:1) at room temperature (5 L × 5 times). The combined extracts were concentrated in vacuo to a brown gum. The dried material (218 g) was subjected to gravity chromatography in a silica gel column (6 × 120 cm) eluting with *n*-hexane (3000 mL), followed by a gradient of *n*-hexane-CH_2_Cl_2_ up to 100% CH_2_Cl_2_ and CH_2_Cl_2_–MeOH up to 50% MeOH (3000 mL each of the solvent mixture). The *n*-hexane/CH_2_Cl_2_ (1:1) fraction (2.2 g) eluted with *n*-hexane/EtOAc (6:1) was subjected to silica gel column separation. Fractions were obtained and combined into two main sub-fractions, A and B, according to a TLC profile. Sub-fraction A was re-purified by reversed-phase HPLC using MeOH/H_2_O (6.5:3.5), 3.5 mL/min, to afford **1** (6.1 mg, *t*_R_ = 27 min), **4** (11.6 mg, *t*_R_ = 23 min), and **5** (11.6 mg, *t*_R_ = 21 min). Sub-fraction B was re-purified by reversed-phase HPLC using MeOH/H_2_O (3:2), 3.5 mL/min, to afford **2** (10 mg, *t*_R_ = 28 min) and **3** (7.5 mg, *t*_R_ = 29.5 min). The *n*-hexane/CH_2_Cl_2_ (1:2) fraction (1.4 g) was subjected to silica gel column chromatography eluted by *n*-hexane/EtOAc (5:1) that afforded the main sub-fraction C. Sub-fraction C was re-purified by reversed-phase HPLC using MeOH/H_2_O (1:1), 3 mL/min, to afford **6** (9.0 mg, *t*_R_ = 31 min), **7** (11.2 mg, *t*_R_ = 32 min), and **8** (14.1 mg, *t*_R_ = 37 min).

*2S,16:7R,12S-Diepoxy-11α-hydroxy-8β-methoxy-16-keto-cembra-1E,3E-diene* (*Sarcoehrenbergilid A,* **1**): white crystals; ${\left[\alpha \right]}_{25}^{D}$ −6.9 (*c* 0.0023, CHCl_3_); FT-IR (KBr) ν_max_: 3450, 2933, 1745, 1455, and 1220 cm^−1^; ^1^H and ^13^C NMR data, see Table 1; HRESI-FTMS *m*/*z* 387.2142 [100, (M + Na)^+^]; (calcd. 364.2250, for C_21_H_32_O_5_).

*2S,16:8S,11S-Diepoxy-7β,12α-dihydroxy-16-keto-cembra-1E,3E-diene* (*Sarcoehrenbergilid B,* **2**): white powder; ${\left[\alpha \right]}_{25}^{D}$ −6.6 (*c* 0.003, CHCl_3_); FT-IR (KBr) ν_max_: 3447, 2933, 1747, 1457, and 1219 cm^−1^; ^1^H and ^13^C NMR data, see Table 1; HRESI-FTMS *m*/*z* 373.1986 [100, (M + Na)^+^]; (calcd. 350.2093, for C_20_H_30_O_5_).

*2S,16:7R,11R-Diepoxy-8β,12β-dihydroxy-16-keto-cembra-1E,3E-diene* (*Sarcoehrenbergilid C,***3**)*:* white amorphous powder; ${\left[\alpha \right]}_{25}^{D}$ −3.7 (*c* 0.0027, CHCl_3_); FT-IR (KBr) ν_max_: 3447, 2933, 1747, 1451, and 1231 cm^−1^;^1^H and ^13^C NMR data, see Table 1; HRESI-FTMS *m*/*z* 373.1985 [100, (M + Na)^+^]; (calcd. 350.2093 for C_20_H_30_O_5_).

#### X-ray Crystallography Data

Data collection was performed with a Bruker SMART-APEX II ULTRA CCD area detector with graphite monochromated Cu Kα radiation (λ = 1.54178 Å) at the Center for Analytical Instrumentation, Chiba University, Japan. The structure was solved by direct methods using SHELXS-97 [26]. Refinements were performed with SHELXL-2013 [27] using full-matrix least squares on *F*^2^. All non-hydrogen atoms were refined anisotropically. All hydrogen atoms were placed in idealized positions and refined as riding atoms isotropically. Crystal data: C_21_H_32_O_5_, *M* = 364.46, monoclinic, crystal size, 0.30 × 0.30 × 0.10 mm^3^, Space group *P*21, *Z* = 2, crystal cell parameters *a* = 5.74330 (10) Å, *b* = 16.8653 (3) Å, *c* = 10.2383 (2) Å, α = 90˚, β = 95.7012 (7)˚, γ = 90˚, *V* = 986.80 (3) Å^3^, *F*(000) = 396, *Dc* = 1.227 Mg/m^3^, *T* = 173 K, 12540 reflections measured, 3480 independent reflections [*R*_(int)_ = 0.0216], final *R* indices [*I* > 2.0σ(*I*)], *R*_1_ = 0.0307, *wR*_2_ = 0.0844; final *R* indices (all data), *R*_1_ value = 0.0308, *wR*_2_ = 0.0845, Flack parameter [24]: 0.09 (3). CCDC-1532555 contains the supplementary crystallographic data for this paper. The data can be obtained free of charge from The Cambridge Crystallographic Data Centre via http://www.ccdc.cam.ac.uk/conts/retrieving.html (or from the CCDC, 12 Union Road, Cambridge CB2 1EZ, UK; Fax: +44 1223 336033; E-mail: deposit@ccdc.cam.ac.uk).

### 3.4. Cell Culture

All materials and reagents for the cell cultures were purchased from Lonza (Verviers, Belgium). Human cancer cell lines of non-small cell lung adenocarcinoma (A549), colon adenocarcinoma (Caco-2), and hepatocellular carcinoma (HepG2) (ATCC^®^) were maintained as monolayer culture in Dulbecco’s modified Eagle’s medium (DMEM) supplemented with 10% FBS, 4 mM l-glutamine, 100 U/mL penicillin, and 100 µg/mL streptomycin sulfate. Monolayers were passaged at 70–90% confluence using a trypsin-EDTA solution. All cell incubations were maintained in a humidified CO_2_ incubator with 5% CO_2_ at 37 °C.

### 3.5. Cell Proliferation Assay

Anti-proloiferative studies were performed using a modified MTT (3-[4,5,[4,5-dimethylthiazole-2-yl]-2,5-diphenyltetrazolium bromide) assay based on a previously published method [28,29]. Appropriate cell densities of exponentially growing A549, Caco-2, or HepG2 cells (5000–10000 cells/well) were seeded onto 96-well plates. After a 24 h incubation period with 5% CO_2_ at 37 °C, stock test compounds (**1**–**8**) dissolved in dimethyl sulfoxide (DMSO) were added at concentrations of 100, 50, 25, 12.5, and 6.25 µM in culture medium (final DMSO concentration in medium = 0.1%, by volume). After 48 h of incubation, MTT solution in PBS (5 mg/mL) was added to each well, after which the incubation was resumed for a further 90 min. The formation of intracellular formazan crystals (mitochondrial reduction product of MTT) was confirmed by a phase contrast microscopic examination. At the end of the incubation period, the medium was removed, and 100 µL of DMSO was added to each well to dissolve formed formazan crystals with shacking for 10 min (200 rpm). Dissolved crystals were quantified by reading the absorbance at 492 nm (OD) on a microplate reader (Sunrise™ microplate reader, Tecan Austria Gmbh, Grödig, Austria) and were used as a measure of cell proliferation. 

### 3.6. Anti-Proliferation Quantitative Analysis

Cell proliferation was determined by comparing the average OD values of the control wells with those of the samples (quadrate to octuplet treatments), both represented as % proliferation [control proliferation (0.1% DMSO only) = 100%]. The IC_50_ values (concentration of sample causing 50% loss of cell proliferation of the vehicle control) were calculated using the concentration-response curve fit to the non-linear regression model using GraphPad Prism^®^ v6.0 software (GraphPad Software Inc., San Diego, CA, USA).

### 3.7. Computational Methodology

#### 3.7.1. Density Functional Theory Calculations

The conformational structures for **1**–**3** were generated using Omega2 software (version 2.5.1.4, OpenEye Scientific Software, Santa Fe, NM, USA) [30]. In the conformational search, the energy window value was set to 10 kcal/mol and all stereogenic centers were considered; other parameters were set to default. The geometry of each generated conform was then energetically optimized at the B3LYP/6-31G* level of theory using Gaussian09 software (Revision E.01, Gaussian, Inc., Wallingford, CT, USA) [31]. All optimized conformers were subjected to a vibrational frequency calculation to confirm the minimum energy states and the corresponding free energies were obtained. The relative Boltzmann population of each conformer was then valued at 298 K. Optical rotations were predicted at the same level of theory.

#### 3.7.2. Molecular Docking Studies

Molecular docking in the ATP binding site of EGFR kinase domain was performed using AutoDock 4.2 software (version 4.2, The Scripps Research Institute, La Jolla, CA, USA) [32]. The crystal structure of the EGFR kinase domain complexed with erlotinib (PDB code: 1M17 [33]) was taken as the template for all docking calculations. Water molecules were deleted and all missing hydrogen atoms were added based on the protonation state of the protein. The receptor pdbqt file was then prepared according to AutoDock protocol [34]. The grid center was centered on an erlotinib inhibitor, and the grid box size was set to 60 × 60 × 60 points with a grid spacing of 0.375 Å. 

The number of Autodock GA runs was set to 50 and maximum number of energy evaluations was set to 2,500,000. Other AutoDock parameters were set to their default values. 3D structures were constructed and minimized using an MMFF94S force field with the help of SZYBKI software (version 1.9.0.3, OpenEye Scientific Software, Santa Fe, NM, USA) [35]; atomic charges were assigned using a gasteiger method. Prior to the binding energy calculation, all docked complexes were minimized using AMBER14 software (version 14, University of California, San Francisco, CA, USA) [36]. The studied compounds and receptor were described by the general AMBER force field (GAFF) [37] and AMBER force field 14SB [38], respectively. The atomic partial charges of the studied inhibitors were evaluated using the restrained electrostatic potential (RESP) approach at the HF/6-31G* level. For minimization, the truncated Newton linear conjugate gradient method with LBFGS preconditioning was used. The convergence criterion for the energy gradient was 10^−9^ kcal/mol·Å. A cutoff value of 999 Å and a generalized Born solvent model were used. On the basis of the minimized complex structures, the binding energies were calculated using the molecular mechanical–generalized Born surface area (MM-GBSA) approach. 

The estimated MM-GBSA binding energies were correlated to experimental binding energies, which were calculated based on A549 inhibitory constants using the following equation: Δ*G*_exp_ = RT ln(IC_50_), where IC_50_ in μM and *T* = 298.15 K. To assess the compound **8** stability inside the EGFR active site, a short molecular mechanical simulation of an 8-EGFR complex was first performed using the AMBER software. For the MD simulation, the complex was neutralized and solvated with TIP3P water molecules. The complex was then energetically minimized, heated gradually over a period of 50 ps to 300 K, and equilibrated for 500 ps. The data were then collected over a 2.5 ns simulation with a time step of 2 fs and the SHAKE option to constrain all bonds involving hydrogen atoms was used. 

## 4. Conclusions

Three new (**1**–**3**) and five previously reported (**4**–**8**) terpenoids were isolated and chemically characterized from the Red Sea soft coral *S. ehrenbergi*. The eight identified compounds exhibited differential antiproliferative potential against three human cancer cell lines, with lung A549 cell being the most sensitive to compound treatment. The present study establishes *S. ehrenbergi*as as a new source of sardistrol and a possible antiproliferative candidate against lung cancer. Molecular docking studies are consistent with the binding of **8** to the EGFR kinase domain and the inhibition of cell growth. Molecular docking studies supported high inhibitory activity for **8** versus **6** or **7** with the EGFR kinase domain.

## Figures and Tables

**Table 1:** ^1^H and ^13^C NMR spectral data of **1**–**3** ^a^.

No.	1		2		3	
	δ_H_	δ_C_	δ_H_	δ_C_	δ_H_	δ_C_
1	------	164.3	------	164.6	------	164.4
2	5.50 d (10.1)	81.0	5.49 dd (10.1; 1.8)	81.7	5.51 br d (10.3)	79.6
3	4.94 d (10.1)	119.4	4.99 d (10.1)	120.0	4.87 br d (10.3)	117.8
4	------	141.6	------	142.6	------	146.0
5	2.07 t (13.1) 2.30 m	40.9	2.17 br t (13.1) 2.47 dd (10.1; 13.1)	38.8	2.09 br t (13.0) 2.41 br d (13.0)	40.3
6	2.14 dd (6.8), 1.44 m	27.6	1.66 m; 2.20 m	22.9	1.88 m; 1.58 m	24.9
7	3.24 d (6.9)	78.5	3.44 dd (11.6; 3.6)	69.7	3.01 br d (10.0)	86.8
8	------	80.0	------	73.6	------	69.4
9	1.53 m; 1.91 m	36.4	1.60 m; 2.40 m	36.6	1.88 m; 2.41 br d (13.0)	39.9
10	1.79 m; 2.15 m	28.5	1.61 m; 1.90 m	19.9	1.71 br d (10.9) 1.51 br t (10.9)	23.1
11	3.37 m	79.0	3.37 br d (11.9)	85.1	3.30 br d (10.9)	78.5
12	------	78.3	------	70.1	------	72.8
13	1.45 m; 2.37 m	34.6	1.59 m 1.78 dd (12.5; 3.7)	31.2	1.41 td (13.0; 5.8) 1.78 br t (13.0; 2.0)	35.0
14	1.95 br t (12.2) 2.31 td (12.2, 7.0)	20.8	2.00 br t (12.8) 2.58 td (12.8; 7.0)	21.1	2.17 br t (13.0); 2.67 m	21.1
15	------	122.3	------	122.3	------	122.5
16	------	175.9	------	176.0	------	175.6
17	1.76 s	8.8	1.83 s	8.7	1.82 s	8.7
18	1.86 s	17.0	1.92 br s	16.7	1.85 br s	16.1
19	1.05 s	17.1	1.22 s	22.1	1.15 s	20.0
20	0.98 s	17.6	1.13 s	25.3	1.16 s	23.8
21	3.13 s	49.0				

*J* values (Hz) are in parentheses; ^a^ Recorded in CDCl_3_ and obtained at 600 and 150 MHz for ^1^H and ^13^C NMR, respectively.

**Table 2:** IC_50_ values * of tested compounds against A549, Caco-2, and HepG2 cells.

Compound	A549 IC_50_(µM)	Caco-2 IC_50_(µM)	HepG2 IC_50_(µM)
1	50.1	>100	98.6
2	76.4	>100	>100
3	50.8	>100	53.8
4	91.5	>100	>100
5	62.2	>100	79.3
6	37.0	79.2	70.2
7	43.6	99.2	63.1
8	27.3	>100	56.8
Doxorubicin HCl	0.62	1.40	2.10

* IC_50_ values were obtained by fitting the concentration-response curve to non-linear regression. model on GraphPad ^®^Prism software v 6.0.

## Supplementary Materials

The following are available online at www.mdpi.com/1660-3397/15/6/192/s1, Figures S1–S29: HR-ESI-MS, 1D, and 2D NMR spectra of compounds **1**–**3**, Table S1: Conformer relative free energies with respect to the most stable form, Table S2: Calculated auto-dock and MM/GBSA binding energies (ΔG) for the most and least active compounds complexed with the EGFR kinase domain.
