# Supplementary material for: Cembrene Diterpenoids with Ether Linkages from Sarcophyton ehrenbergi: An Anti-Proliferation and Molecular-Docking Assessment
Source: Mar Drugs. 2017 Jun 21;15(6):192. doi: 10.3390/md15060192 (PMC5484142; doi:10.3390/md15060192)
Supplement: Supplementary file 1 [file marinedrugs-15-00192-s001.pdf]

Supplementary Information

Table S1. Conformer relative free energies with respect to the most stable form.
Table S2. Calculated binding energies (ΔG) for the most and least active compounds.
Figure S1. HR-ESI-FTMS spectrum of Sarcoehrenbergilid A (**1**).
Figure S2. ^{1}H NMR spectrum of Sarcoehrenbergilid A (**1**) in $CDCl_3$.
Figure S3. ^{13}C NMR spectrum of Sarcoehrenbergilid A (**1**) in $CDCl_3$.
Figure S4. DEPT spectrum of Sarcoehrenbergilid A (**1**) in $CDCl_3$.
Figure S5. HSQC spectrum of Sarcoehrenbergilid A (**1**) in $CDCl_3$.
Figure S6. HMBC spectrum of Sarcoehrenbergilid A (**1**) in $CDCl_3$.
Figure S7. NOESY spectrum of Sarcoehrenbergilid A (**1**) in $CDCl_3$.
Figure S8. HR-ESI-FTMS spectrum of Sarcoehrenbergilid B (**2**).
Figure S9. ^{1}H NMR spectrum of Sarcoehrenbergilid B (**2**) in $CDCl_3$.
Figure S10. ^{13}C NMR spectrum of Sarcoehrenbergilid B (**2**) in $CDCl_3$.
Figure S11. ^{1}H-^{1}H COSY spectrum of Sarcoehrenbergilid B (**2**) in $CDCl_3$.
Figure S12. HSQC spectrum of Sarcoehrenbergilid B (**2**) in $CDCl_3$.
Figure S13. HMBC spectrum of Sarcoehrenbergilid B (**2**) in $CDCl_3$.
Figure S14. NOESY spectrum of Sarcoehrenbergilid B (**2**) in $CDCl_3$.
Figure S15. HR-ESI-FTMS spectrum of Sarcoehrenbergilid C (**3**).
Figure S16. ^{1}H NMR spectrum of Sarcoehrenbergilid C (**3**) in $CDCl_3$.
Figure S17. ^{13}C NMR spectrum of Sarcoehrenbergilid C (**3**) in $CDCl_3$.
Figure S18. DEPT spectrum of Sarcoehrenbergilid C (**3**) in $CDCl_3$.
Figure S19. HSQC spectrum of Sarcoehrenbergilid C (**3**) in $CDCl_3$.
Figure S20. HMBC spectrum of Sarcoehrenbergilid C (**3**) in $CDCl_3$.
Figure S21. NOESY spectrum of Sarcoehrenbergilid C (**3**) in $CDCl_3$.
Figure S22. Optimized structure and relative free energy of conformers of **1** with Boltzmann population higher than 1%.
Figure S23. Optimized structure and relative free energy of conformers of **2** with Boltzmann population higher than 1%.
Figure S24. Optimized structure and relative free energy of conformers of **3** with Boltzmann population higher than 1%.
Figure S25. Anti-proliferative A549 response curves with **1-8** based on MTT-reduction assay.
Figure S26. Anti-proliferative Caco-2 response curves with 1-8 based on MTT-reduction assay.
Figure S27. Anti-proliferative HepG2 response curves with **1-8** based on MTT-reduction assay.
Figure S28. Calculated (i) Autodock and (ii) MM/GBSA binding energies of compounds with EGFR kinase domain relative to the experimental binding energies for the tested compounds against A549 cell line.
Figure S29. Hydrogen bond distance between **8** and carboxylate oxygen atom of Asp_{776} inside EGFR active site.

Table S1. Conformer relative free energies with respect to the most stable form.

Compound	Conformer ID	Relative Free Energy ΔG (kj/mol)	Boltzmann Factor[a] $e^{(-\Delta G/RT)}$	Population %	Optical Rotation (degree)[b]
1	1a	0.00	1.00	37.44	-244.3
	1b	+2.8	0.32	11.89	-204.1
	1c	+3.1	0.29	10.74	-16.5
	1d	+3.2	0.27	10.26	67.0
	1e	+4.2	0.18	6.75	33.2
	1f	+4.3	0.17	6.50	-127.6
	1g	+5.3	0.12	4.48	192.7
	1h	+7.0	0.06	2.18	88.2
	1i	+7.4	0.05	1.87	-85.7
					-107.7[c]
2	2a	0.00	1.00	34.21	-88.5
	2b	+0.2	0.94	32.03	-89.5
	2c	+1.5	0.54	18.48	68.8
	2d	+4.4	0.17	5.82	223.6
	2e	+6.6	0.07	2.37	-37.8
	2f	+6.8	0.07	2.24	37.5
	2g	+7.4	0.05	1.70	38.4
					-32.6[c]
3	3a	0.00	1.00	37.62	-162.84
	3b	+0.9	0.69	25.82	65.13
	3c	+4.0	0.20	7.41	-10.52
	3d	+5.5	0.11	4.11	-119.51
	3e	+5.9	0.09	3.47	-10.25
	3f	+6.0	0.09	3.34	-13.39
	3g	+6.0	0.09	3.33	132.21
	3h	+6.7	0.07	2.52	-96.37
	3i	+6.9	0.06	2.35	-40.38
	3j	+8.6	0.03	1.16	8.17
					-49.8[c]

[a]Calculated at 298K.

[b]Calculated at B3LYP/6-31G* level of theory with the polarizable continuum model (PCM) for chloroform.

[c]Boltzmann-weighted mean optical rotation.

Table S2. Calculated auto-dock and MM/GBSA binding energies (ΔG) for the most and least active compounds complexed with the EGFR kinase domain.

Compound	IC_{50} (μM)[a]	ΔG_{exp} (kcal/mol)[b]	ΔG_{calc} (kcal/mol)		Binding Features		
			AutoDock	MM/GBSA	Residue	Type	Length (Å)
Erlotinib	nd	---	-7.23	-47.89	Met_{769}	H-bond	2.01
					Cys_{773}	H-bond	2.05
Afatinib	nd	---	-7.68	-55.90	Met_{769}	H-bond	2.09
					Leu_{694}	H-bond	2.10
					Cys_{773}	H-bond	1.90
					Lys_{692}	H-bond	1.91
Gefitinib	nd	---	-7.69	-50.23	Thr_{766}	H-bond	2.08
					Met_{769}	H-bond	2.24
Doxorubicin	0.62	-8.46	-8.94	-54.72	Lys_{721}	H-bond	1.92
					Thr_{766}	H-bond	1.76
					Met_{769}	H-bond	1.82
					Thr_{830}	H-bond	1.92
					Asp_{831}	H-bond	1.94
8	27.3	-6.22	-8.13	-41.18	Thr_{766}	H-bond	1.79
					Asp_{776}	H-bond	1.62
7	43.6	-5.94	-5.82	-36.52	Thr_{766}	H-bond	1.75
					Thr_{830}	H-bond	1.87
6	37.0	-6.04	-6.14	-38.04	Met_{769}	H-bond	1.82
					Asp_{831}	H-bond	1.65
4	91.5	-5.51	-7.17	-31.81	Lys_{721}	H-bond	1.82
Correlation coefficient (R^2)			0.37[c]	0.96[c]			

[a] IC_{50} Values for the tested compounds against A549 cell line.

[b]Calculated based on the experimental IC_{50}.

[c]Erlotinib, afatinib and gefitinib were not considered in the calculated correlation due to the absence of the corresponding experimental IC_{50}.

Figure S1. HR-ESI-FTMS spectrum of Sarcoehrenbergilid A (**1**) in CDCl3.

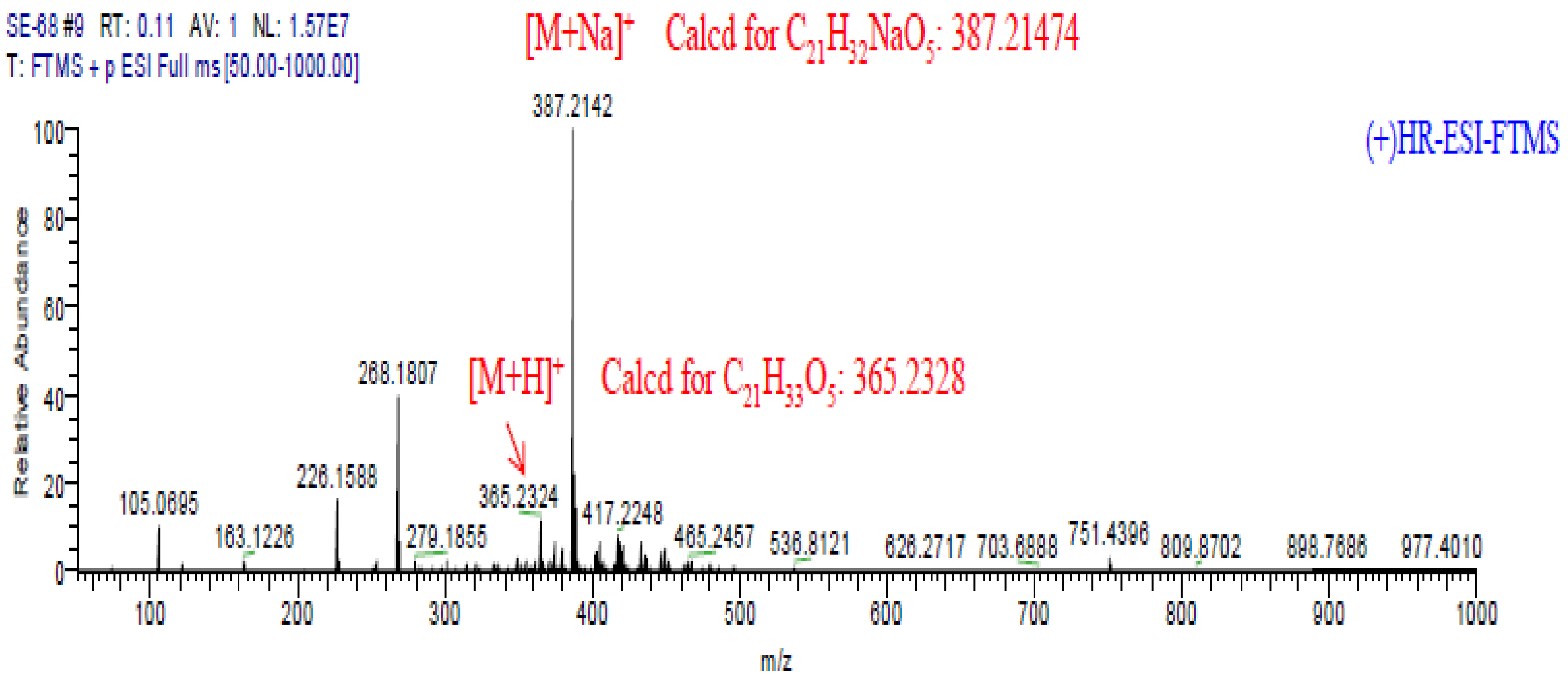

Figure S2. ^{1}H NMR spectrum of Sarcoehrenbergilid A (**1**) in $CDCl_3$.

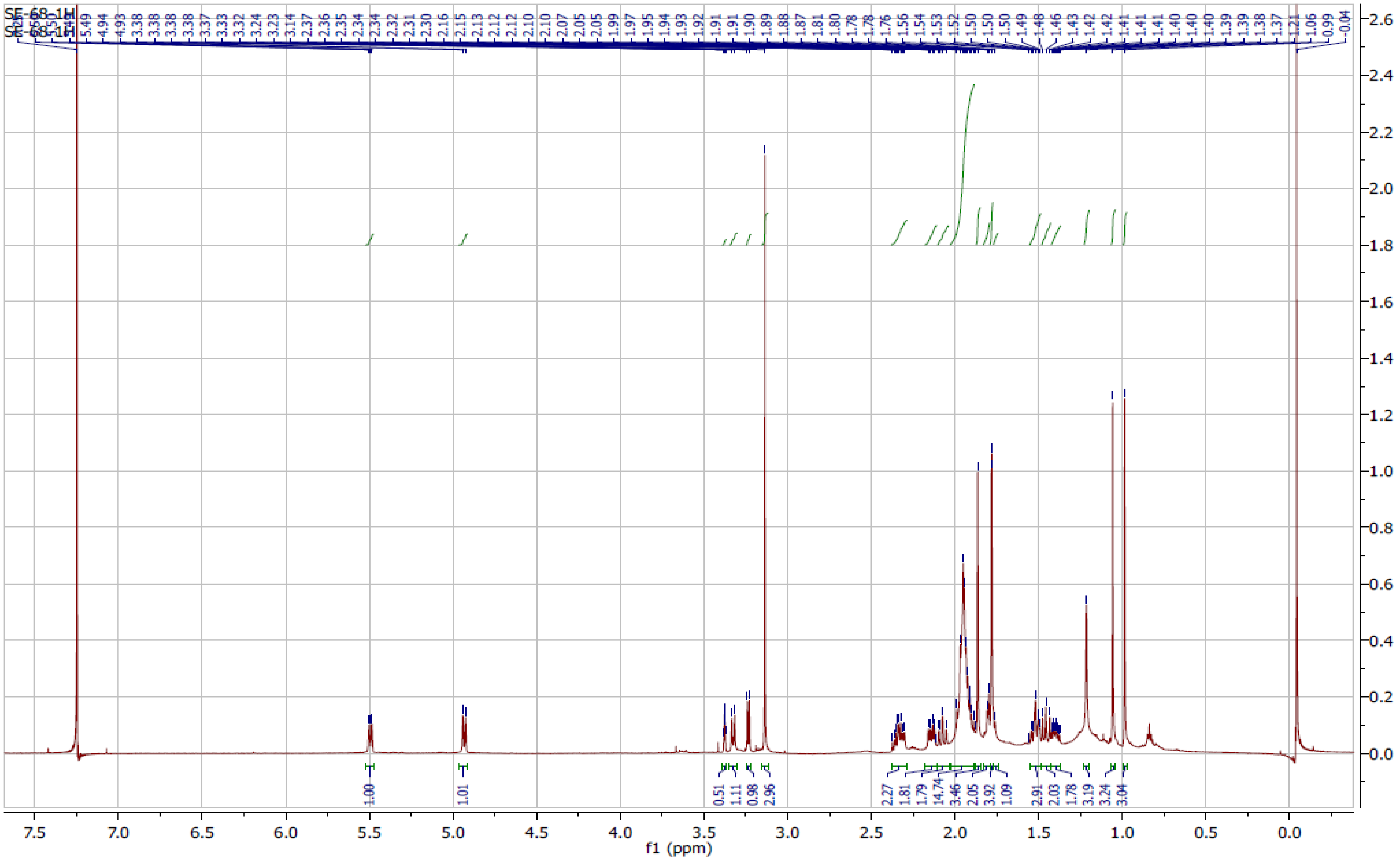

Figure S3. ^{13}C NMR spectrum of Sarcoehrenbergilid A (**1**) in $CDCl_3$.

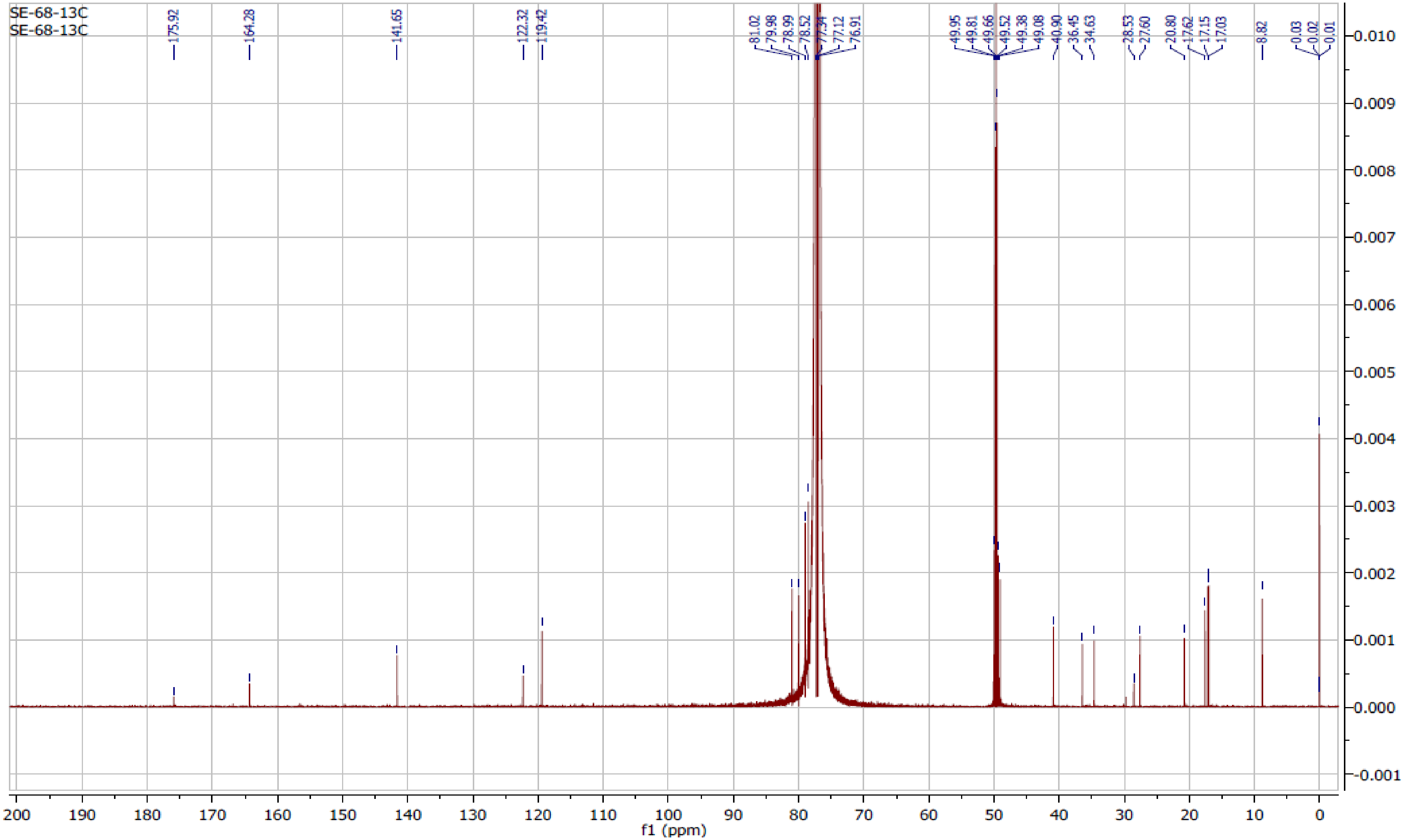

Figure S4. DEPT spectrum of Sarcoehrenbergilid A (**1**) in $CDCl_3$.

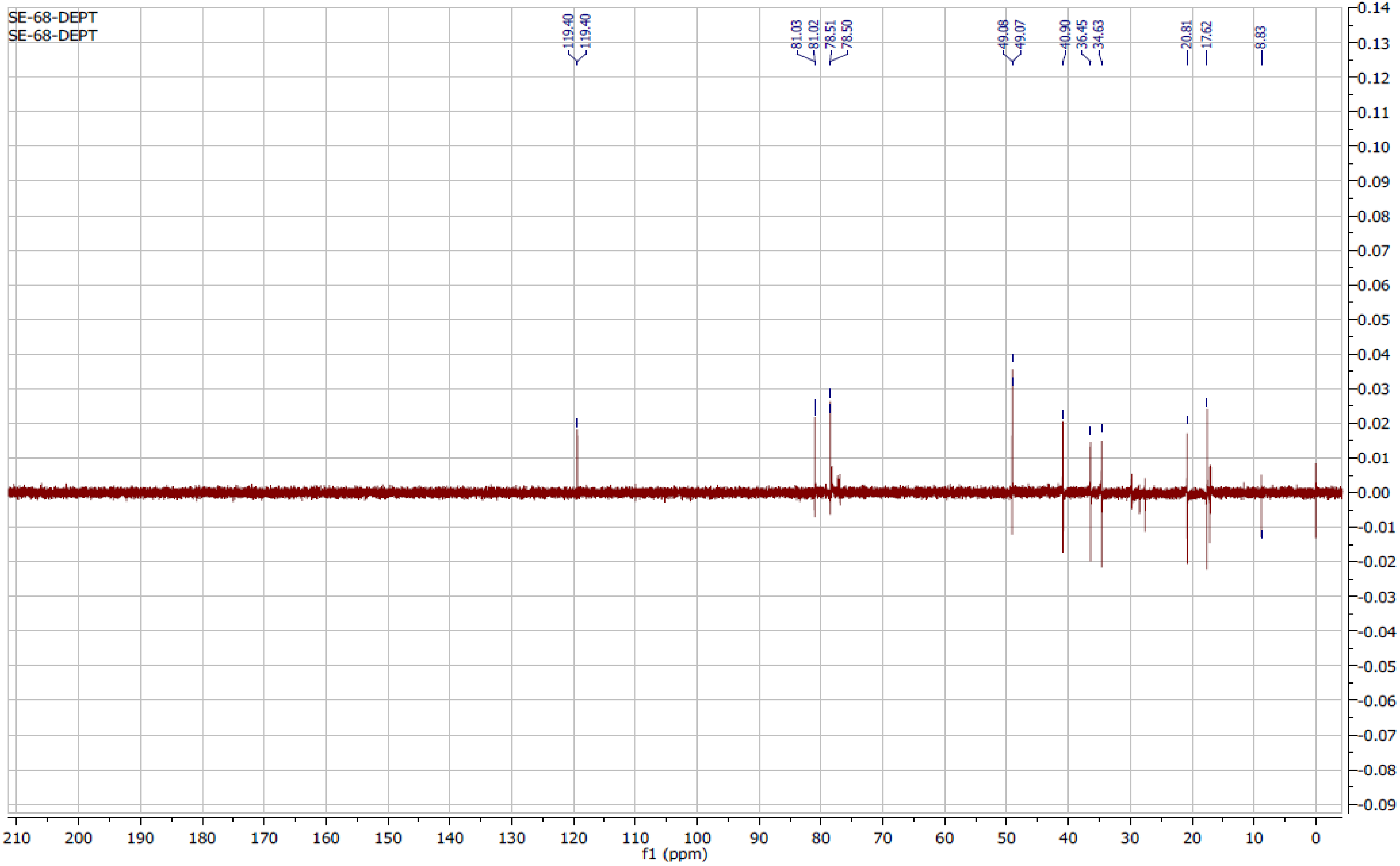

Figure S5. HMBC spectrum of Sarcoehrenbergilid A (**1**) in $CHCl_3$.

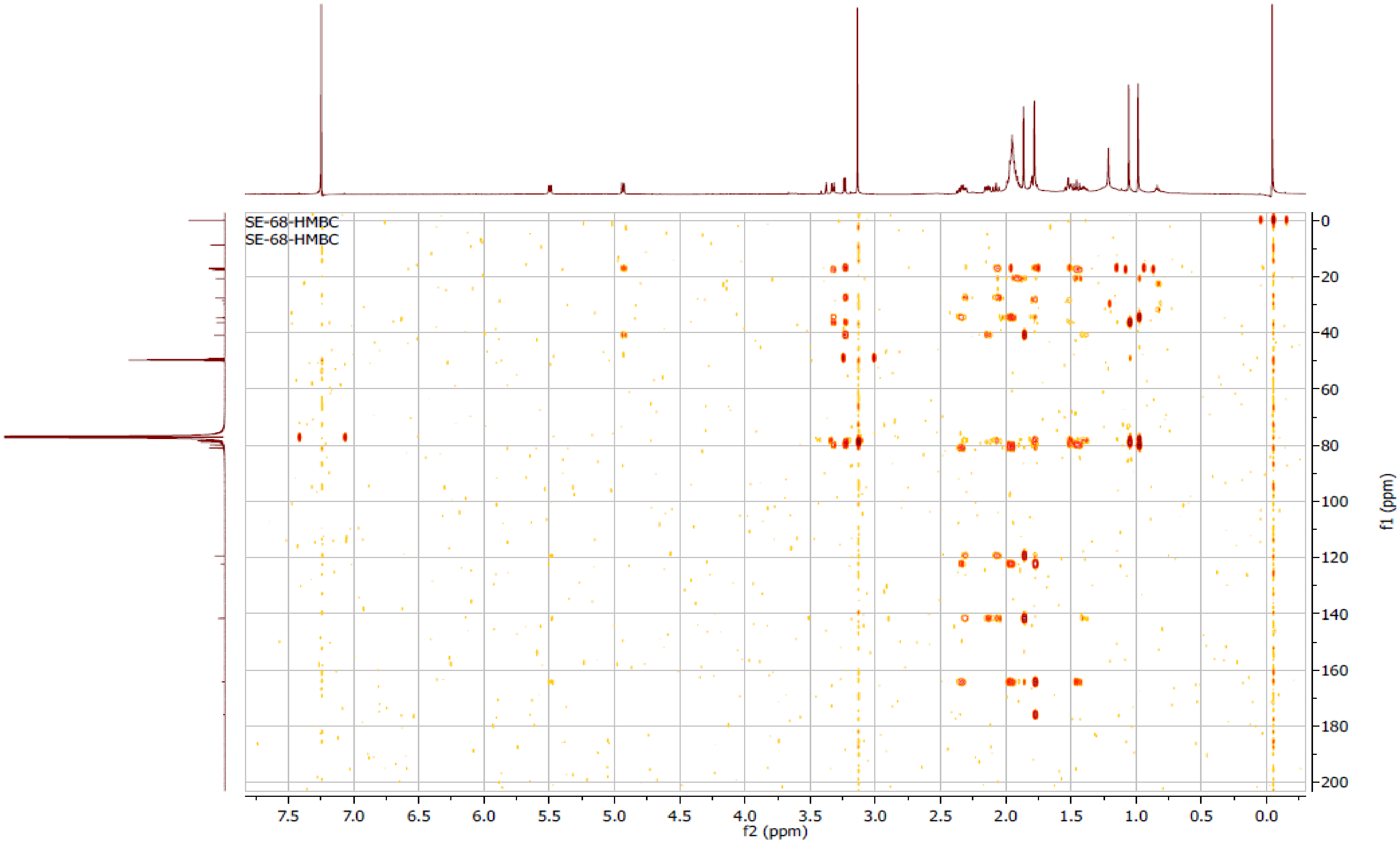

Figure

Fig. S6. ^{1}H, ^{1}H COSY spectrum of Sarcoehrenbergilid A (**1**) in $CDCl_3$.

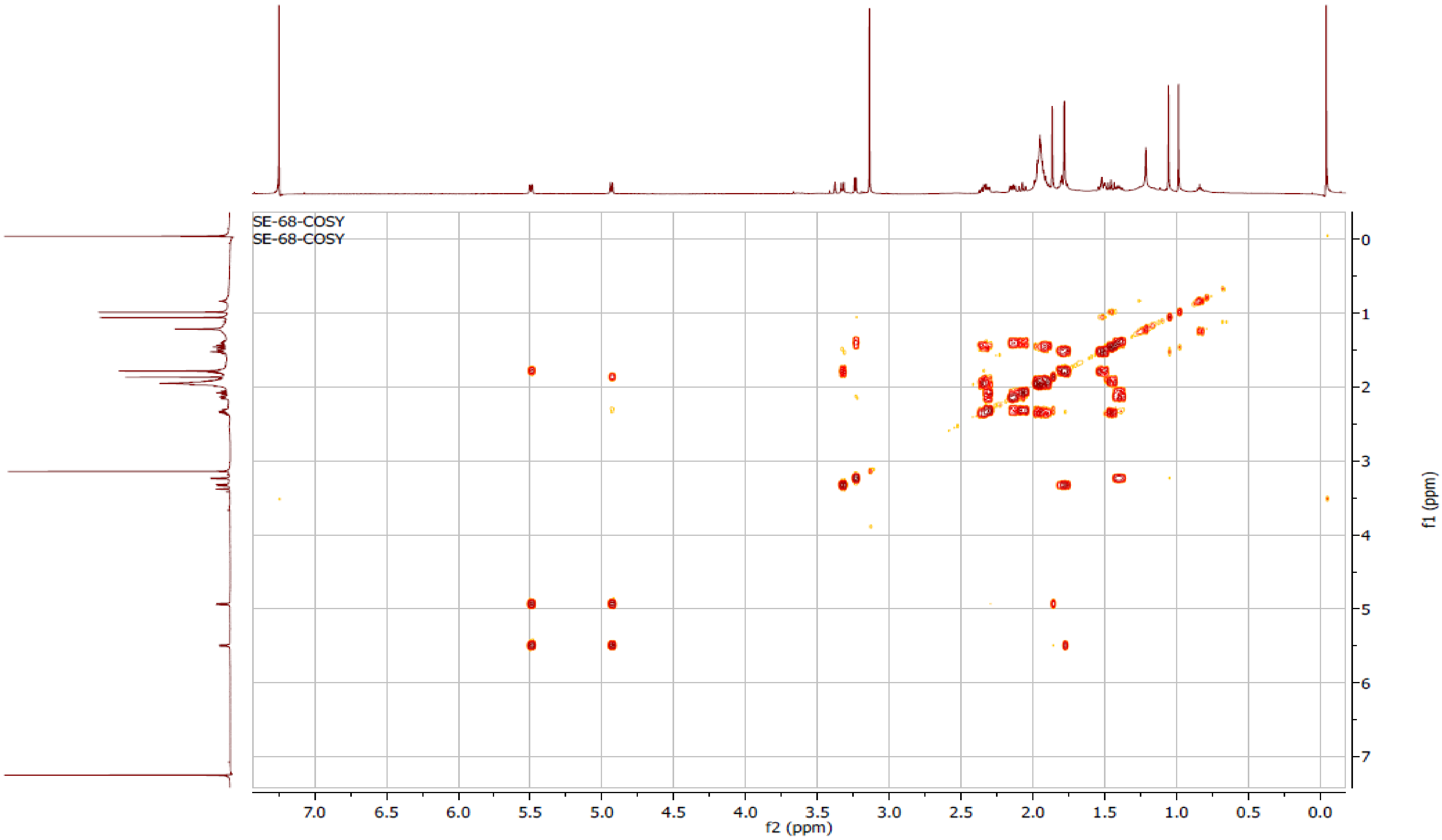

Figure S7. NOESY spectrum of Sarcoehrenbergilid A (**1**) in $CDCl_3$.

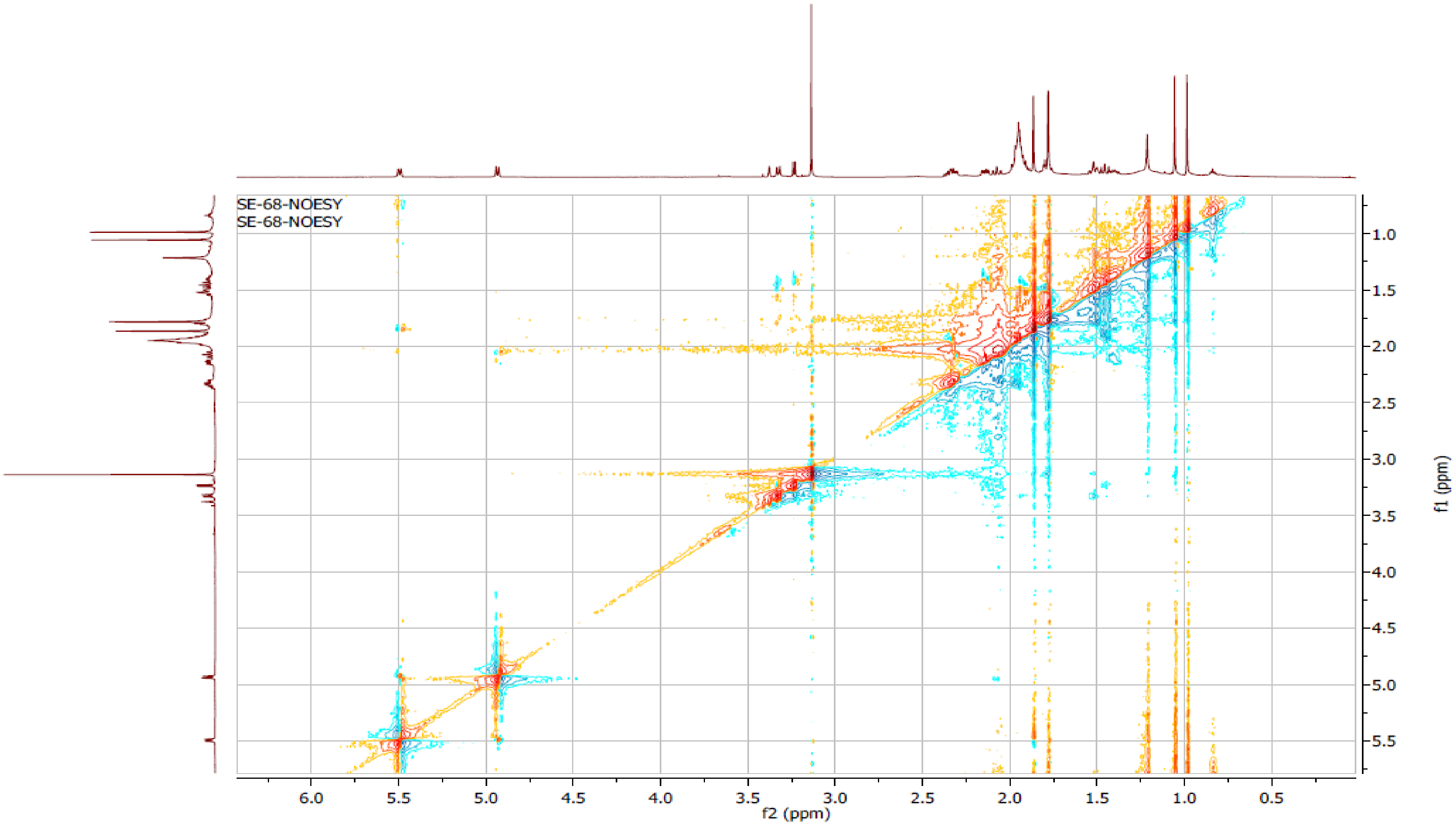

Figure S8. HR-ESI-FTMS spectrum of Sarcoehrenbergilid B (**2**).

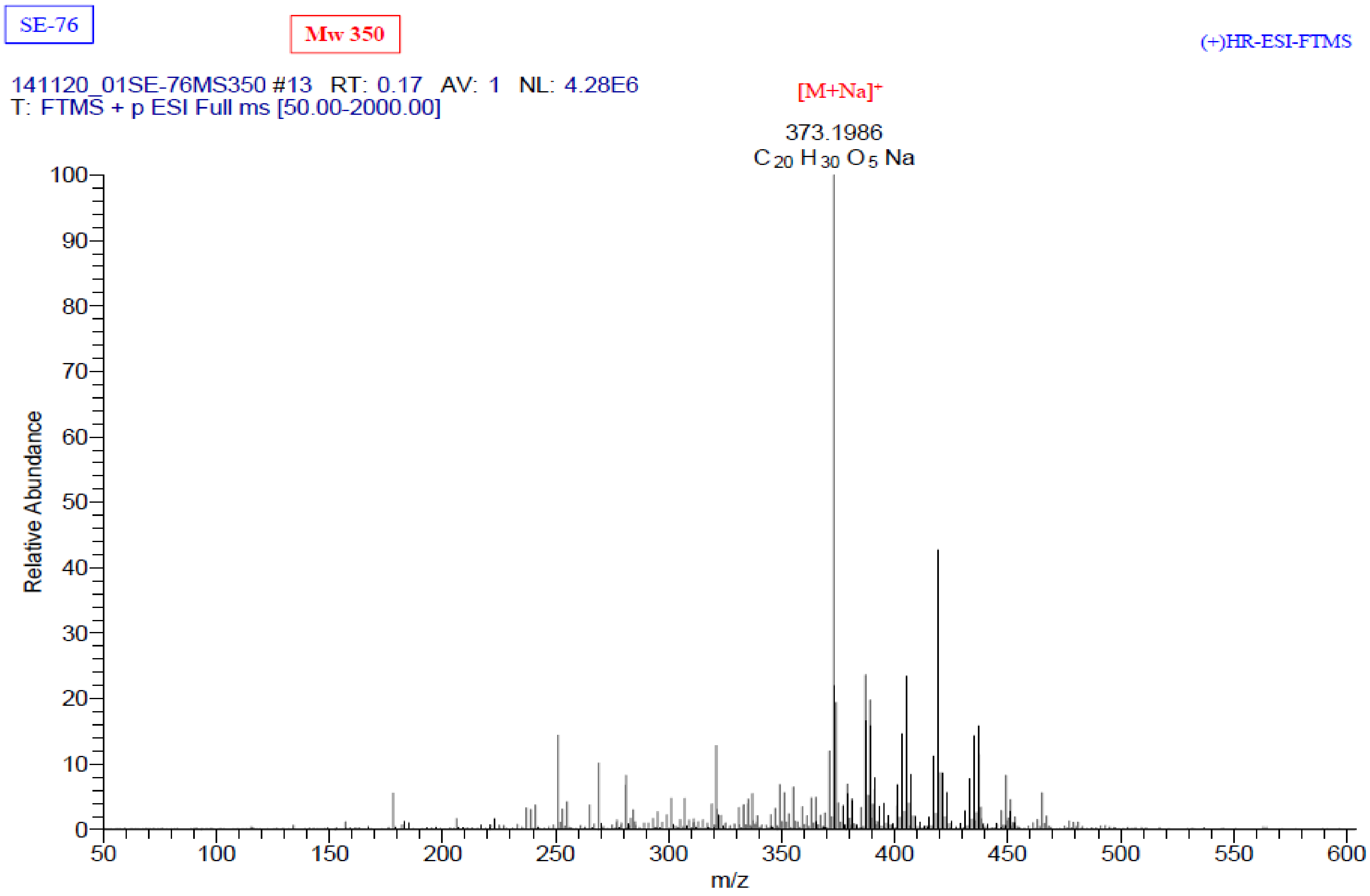

Figure S9. ^{1}H NMR spectrum of Sarcoehrenbergilid B (**2**) in $CDCl_3$.

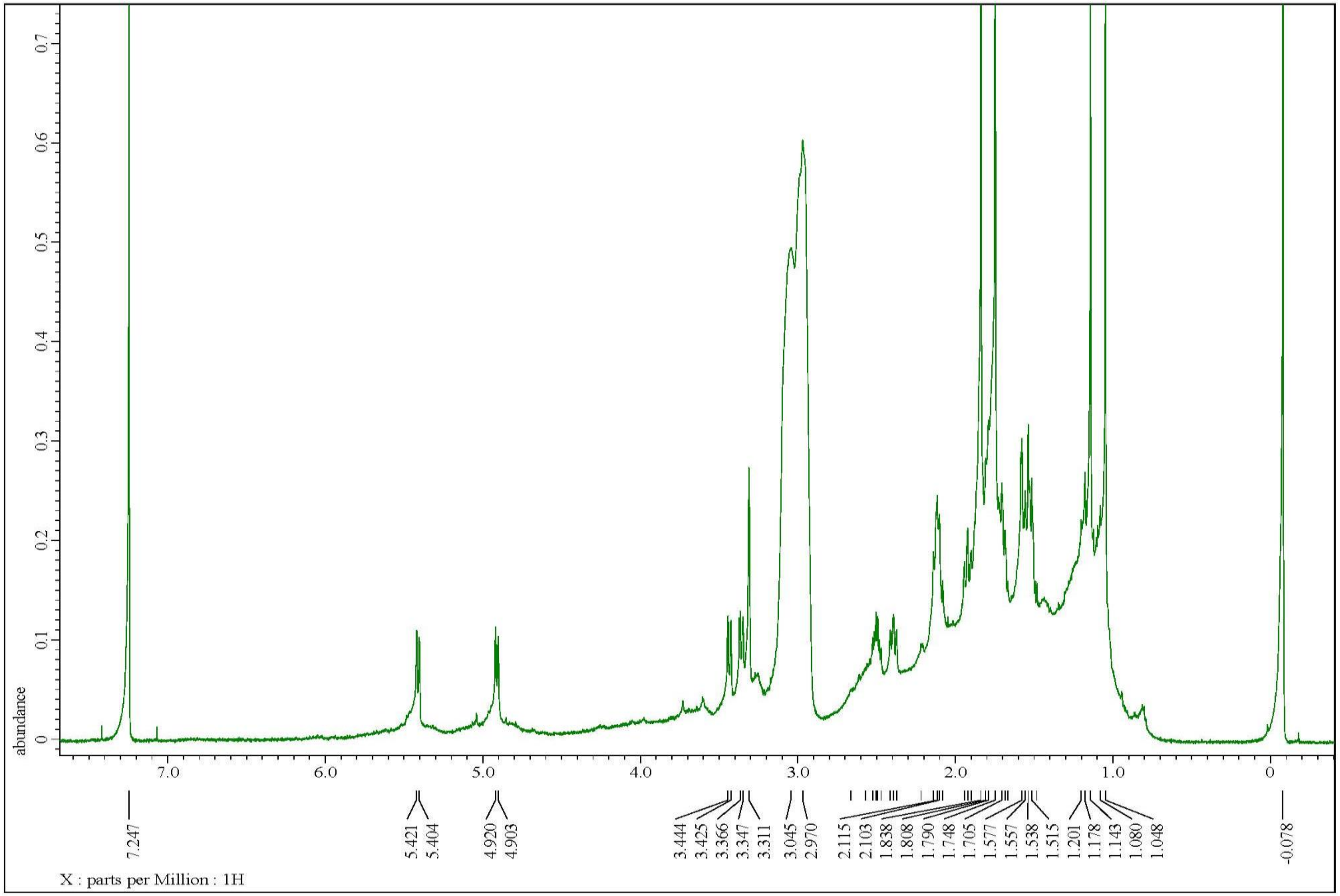

Figure S10. ^{13}C NMR spectrum of Sarcoehrenbergilid B (**2**) in $CDCl_3$.

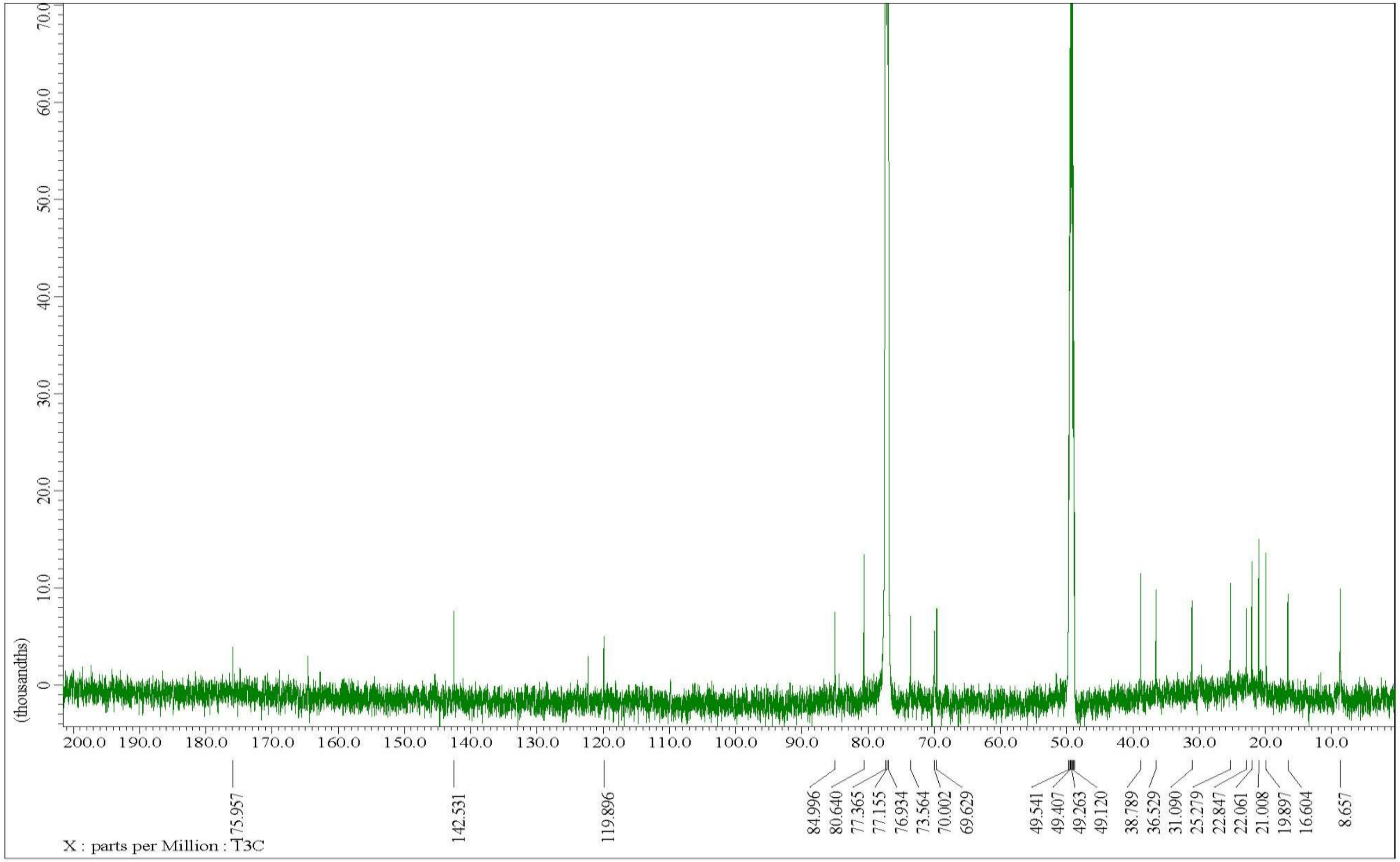

Figure S11. ^1H-^1H COSY spectrum of Sarcoehrenbergilid B (**2**) in $CDCl_3$.

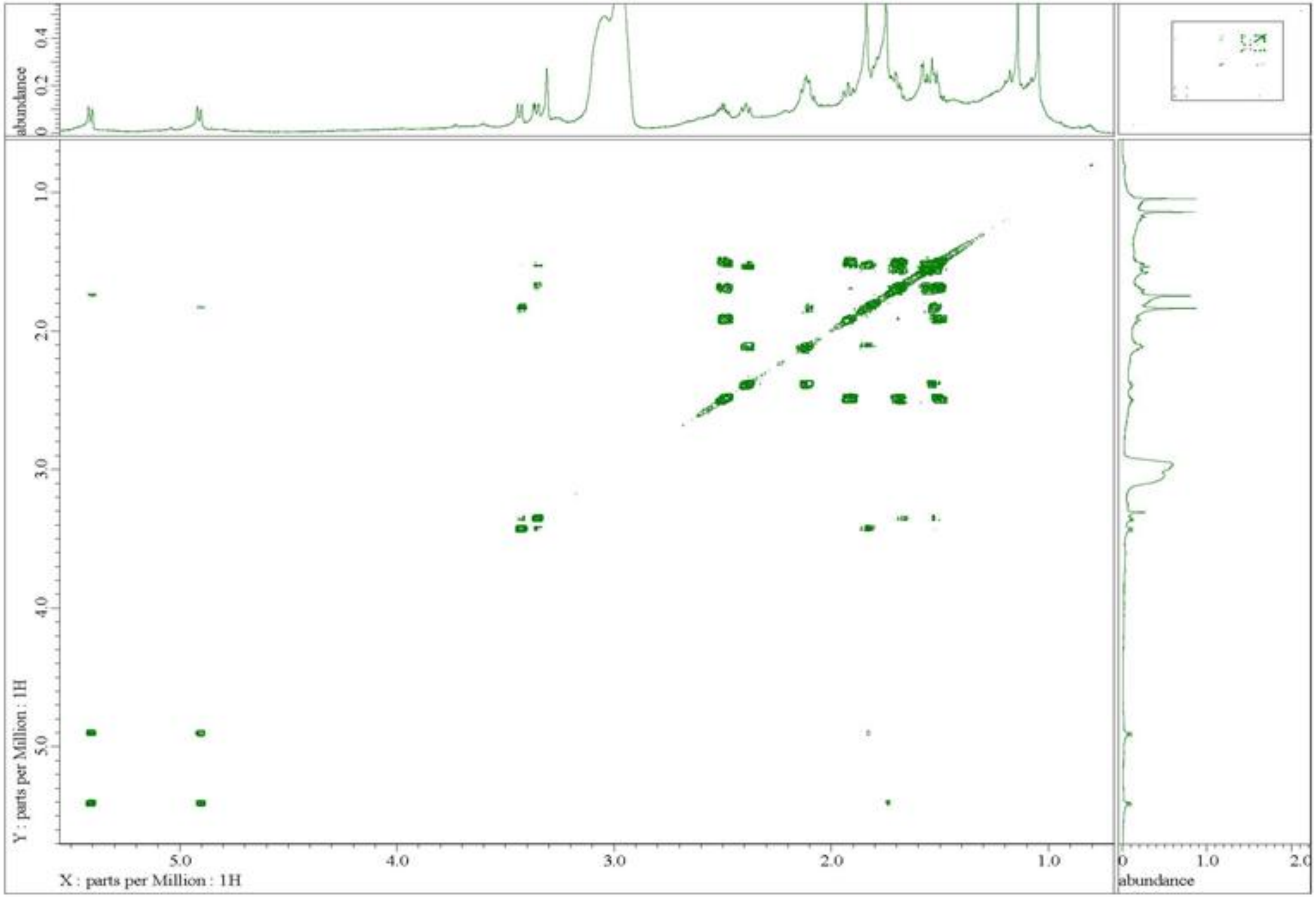

Figure S12. HSQC spectrum of Sarcoehrenbergilid B (**2**) in $CDCl_3$.

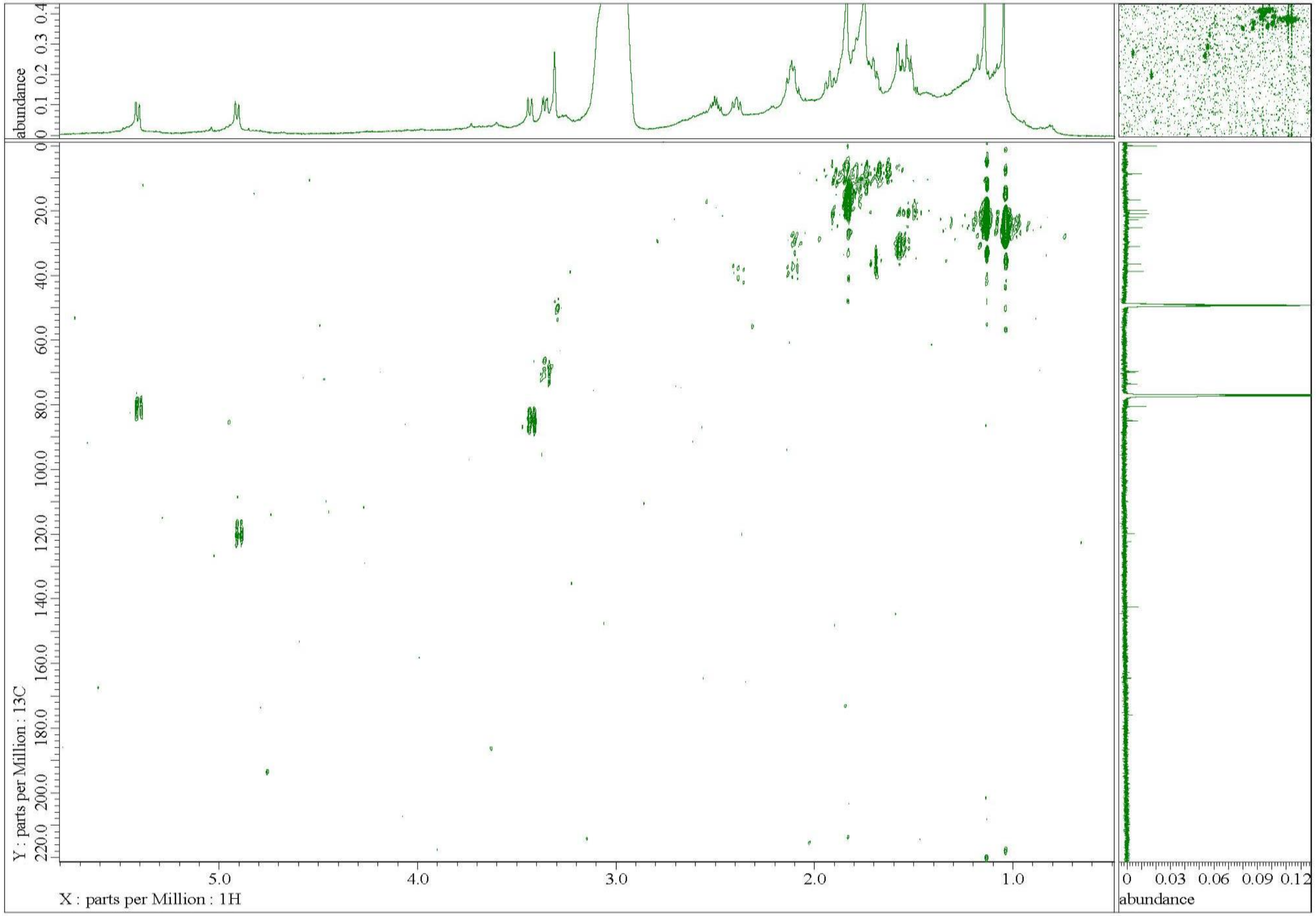

Figure S13. HMBC spectrum of Sarcoehrenbergilid B (**2**) in $CDCl_3$.

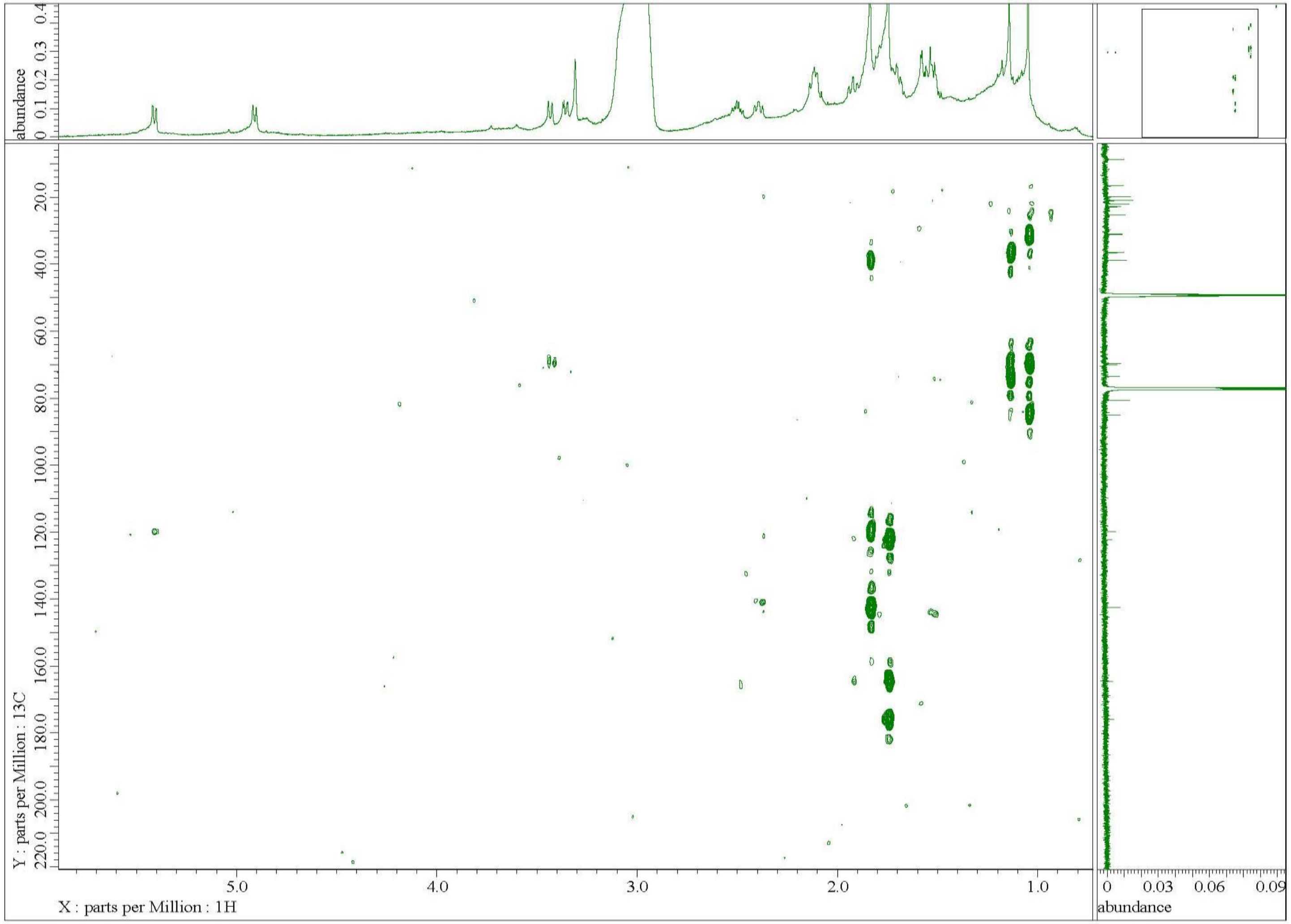

Figure S14. NOESY spectrum of Sarcoehrenbergilid B (**2**) in $CDCl_3$.

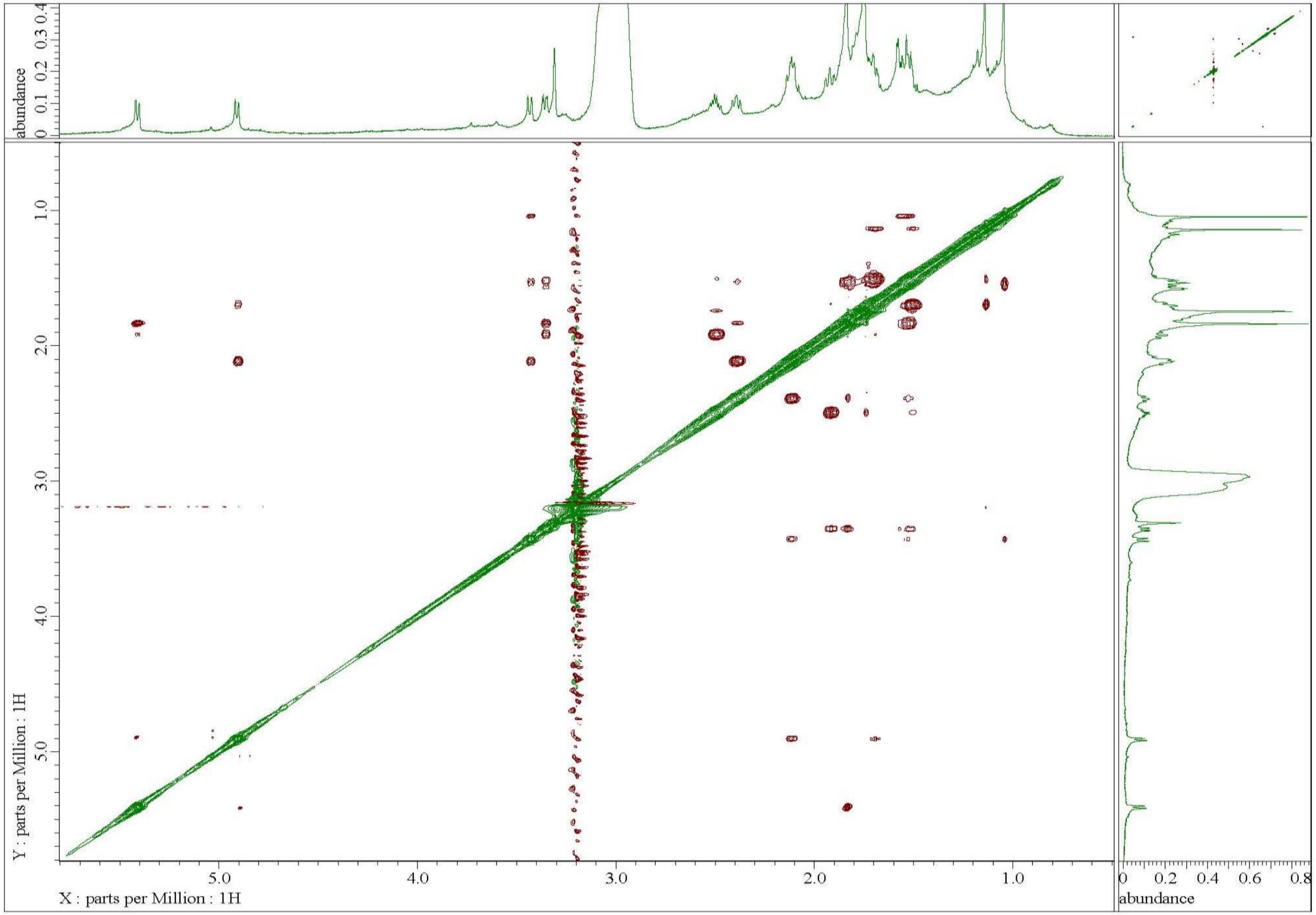

Figure S15. HR-ESI-FTMS spectrum of Sarcoehrenbergilid C (**3**).

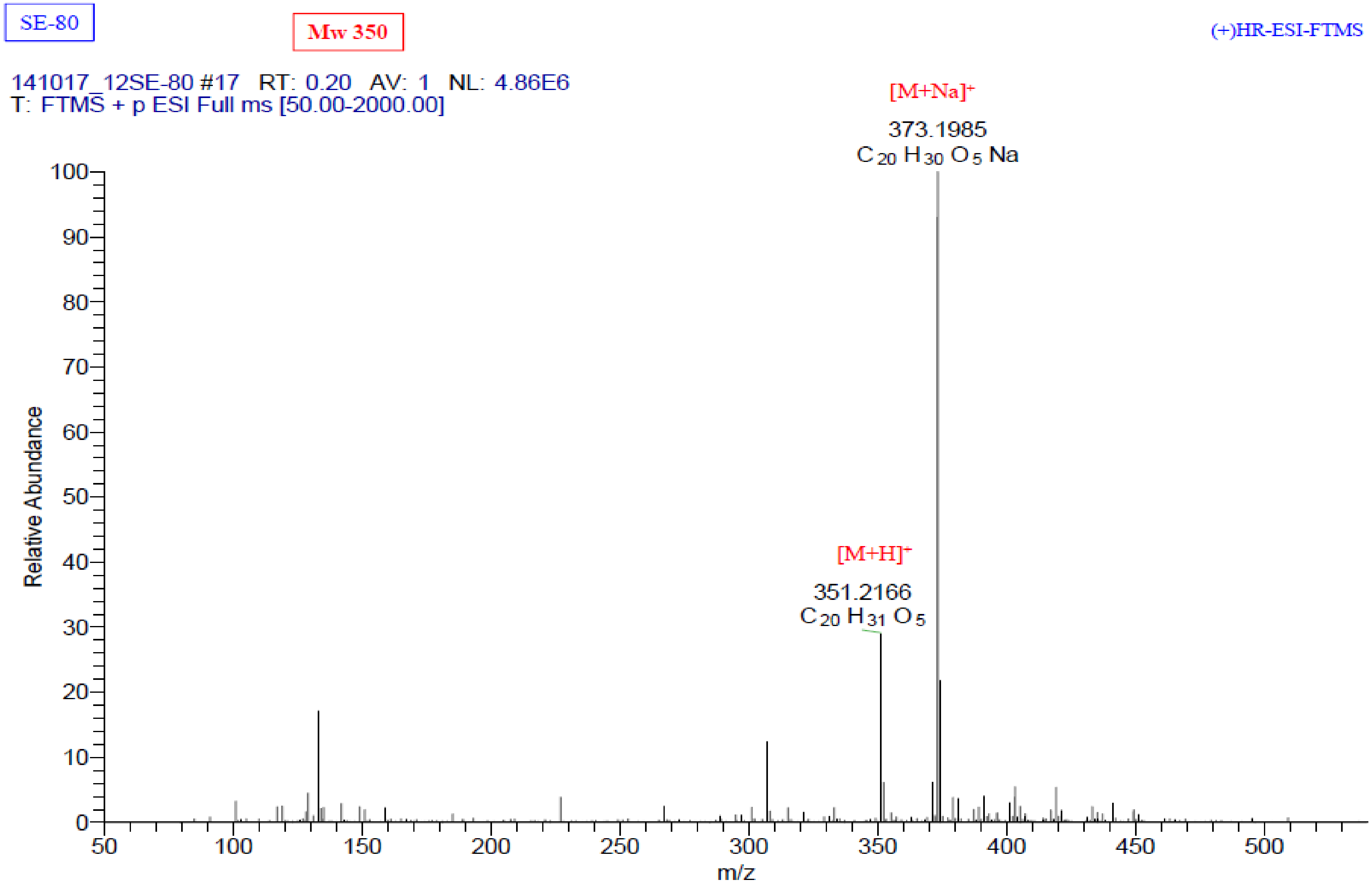

Figure S16. ^{1}H NMR spectrum of Sarcoehrenbergilid C (**3**) in $CDCl_3$.

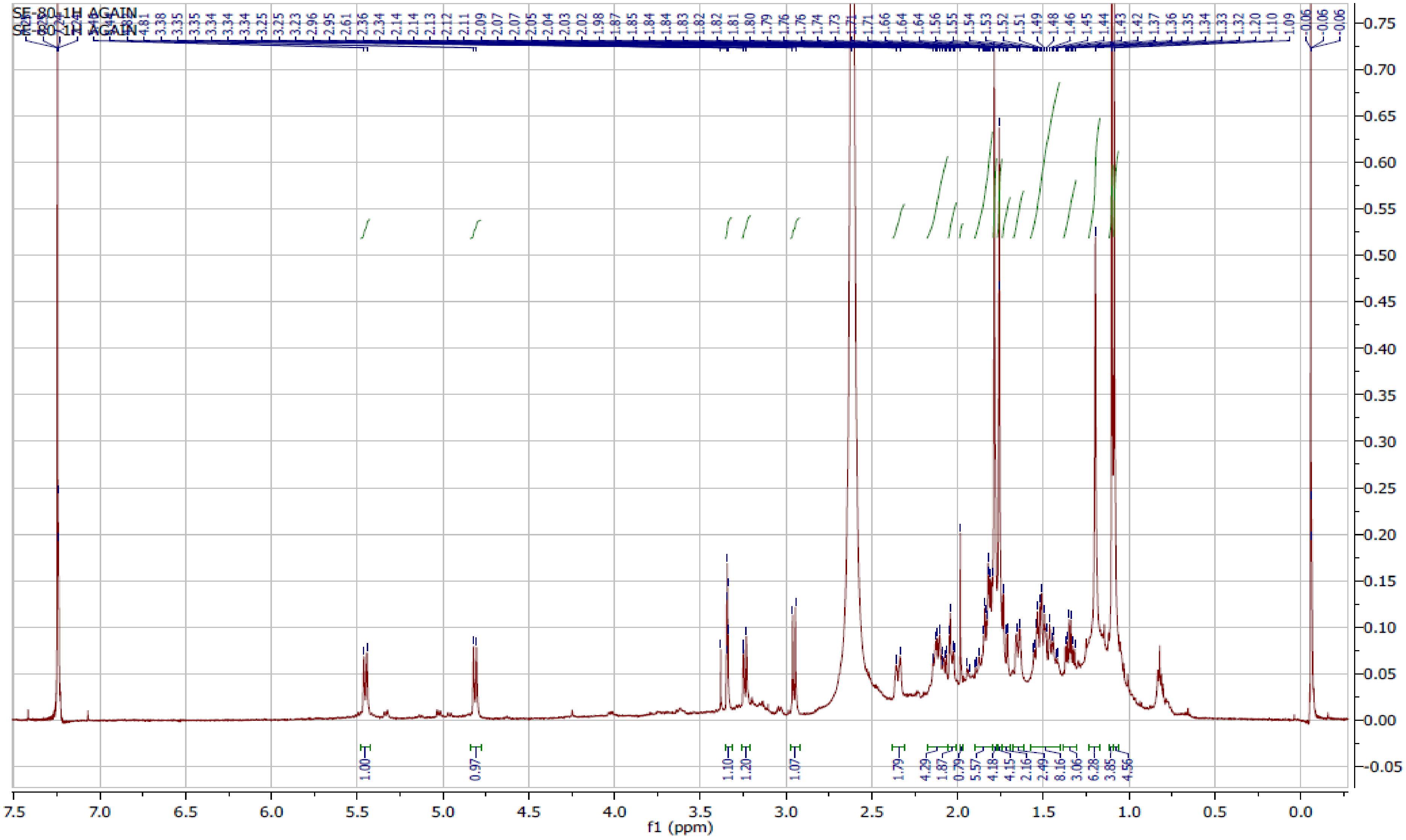

Figure S17. ^{13}C NMR spectrum of Sarcoehrenbergilid C (**3**) in $CDCl_3$.

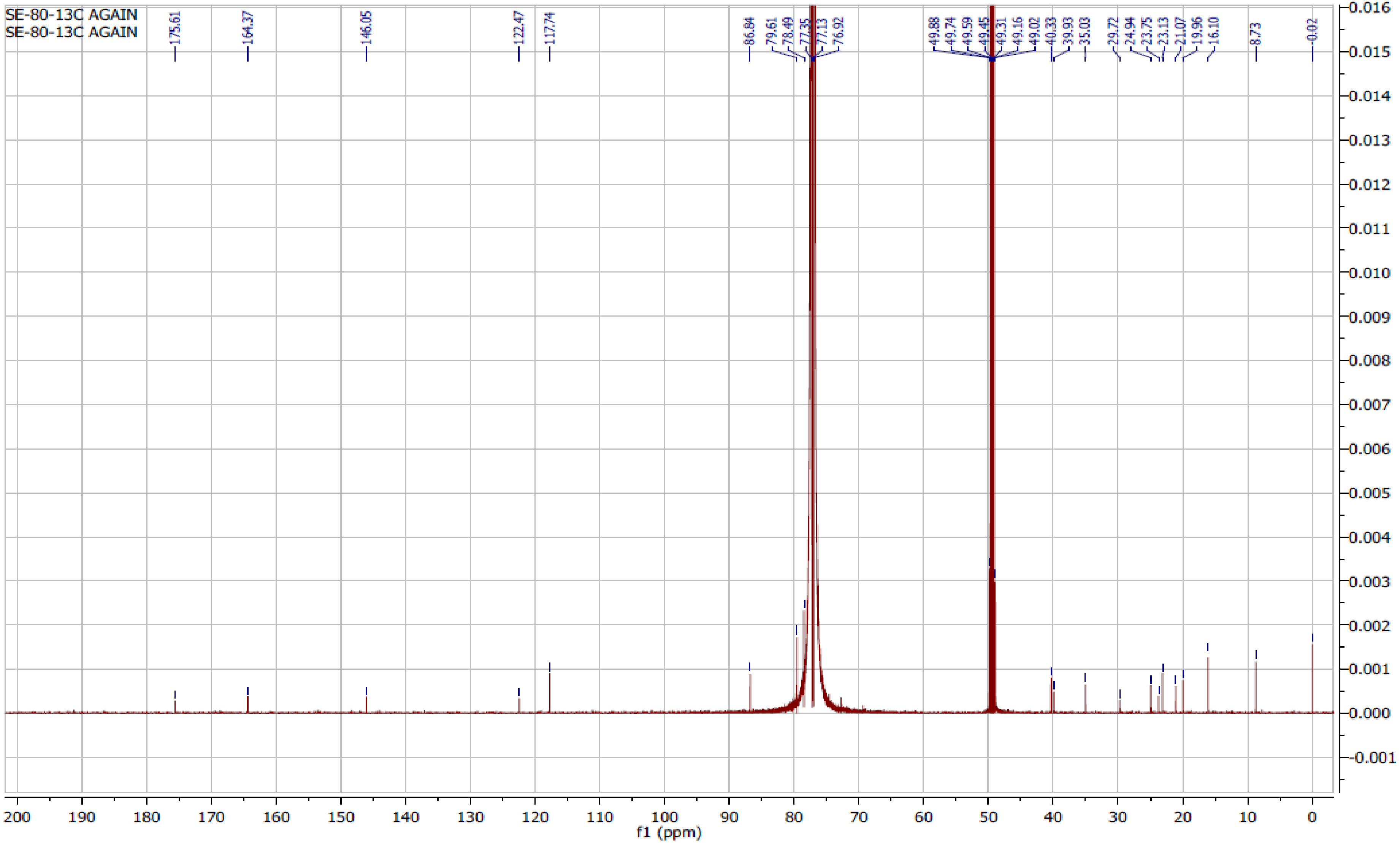

Figure S18. DEPT spectrum of Sarcoehrenbergilid C (**3**) in $CDCl_3$.

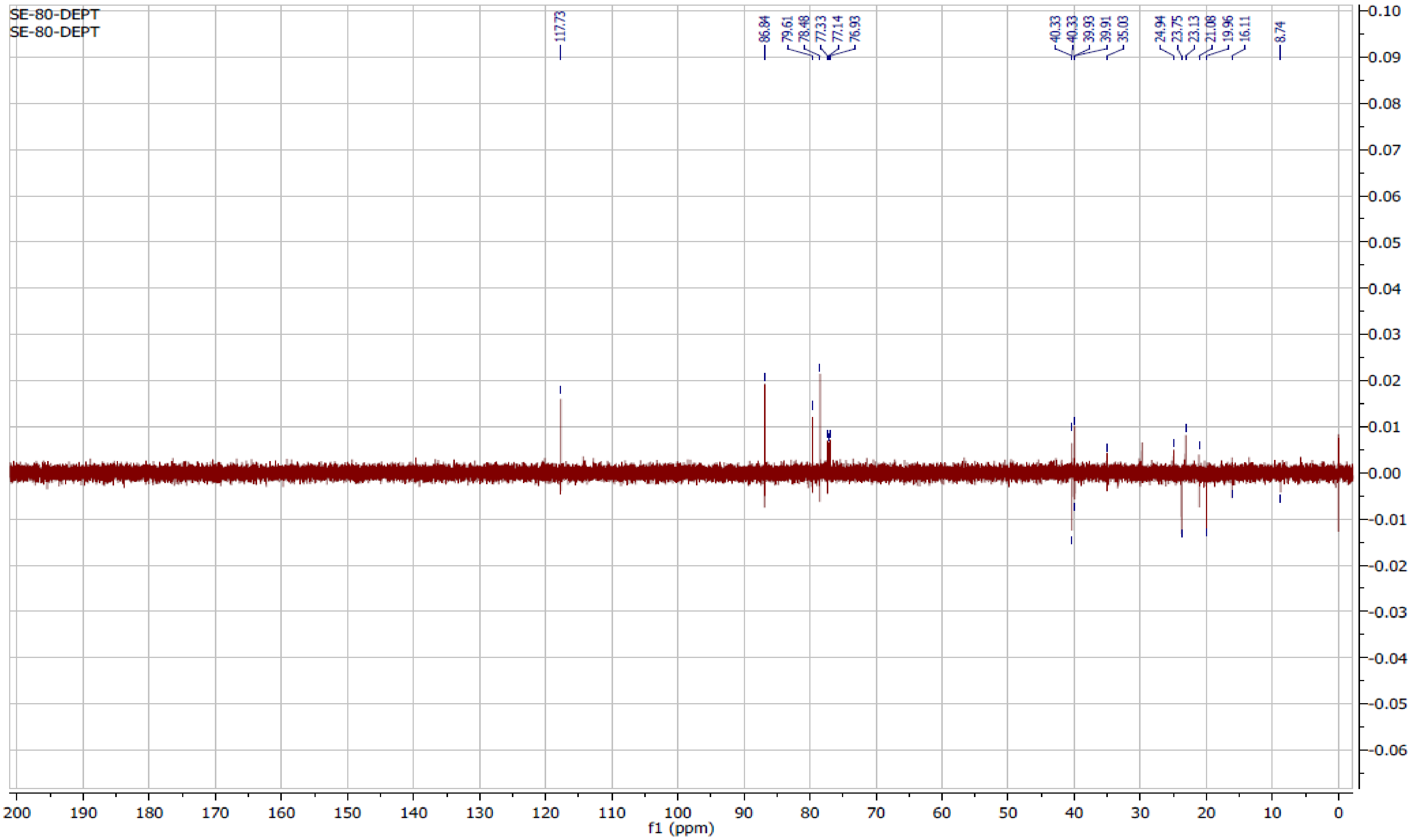

Figure S19. HSQC spectrum of Sarcoehrenbergilid C (**3**) in $CDCl_3$.

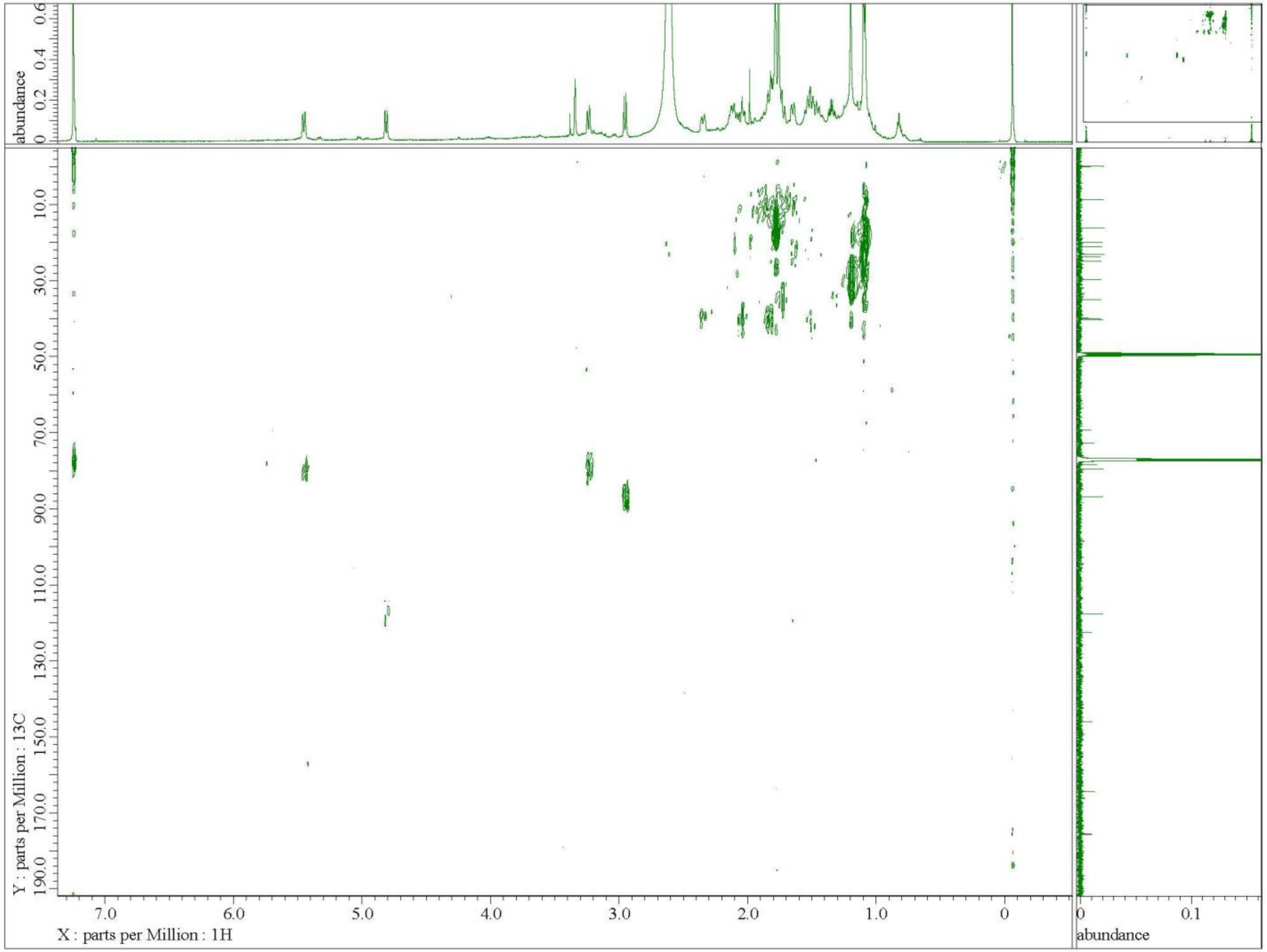

Figure S20. HMBC spectrum of Sarcoehrenbergilid C (**3**) in $CDCl_3$.

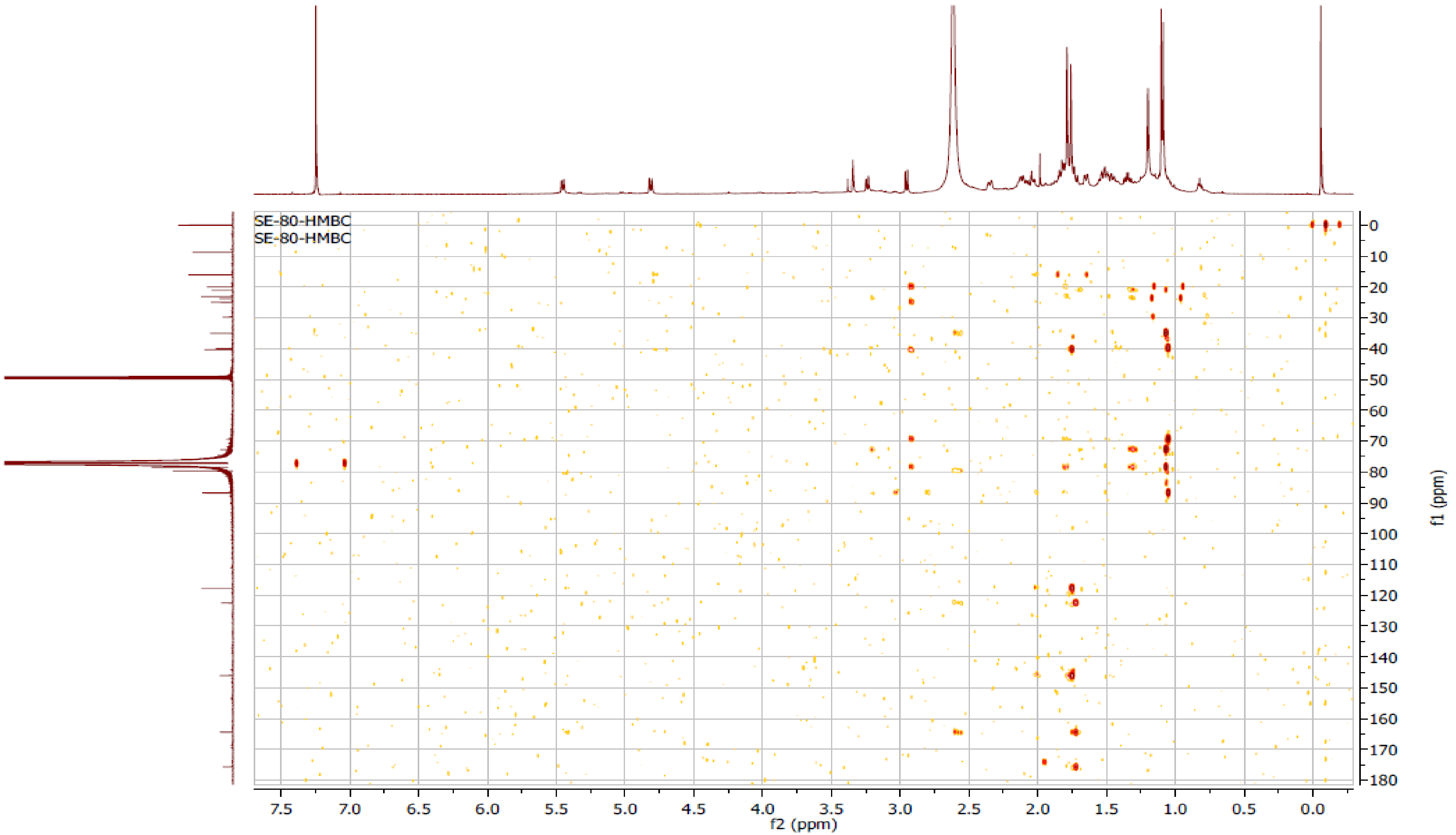

Figure S21. NOESY spectrum of Sarcoehrenbergilid C (**3**) in $CDCl_3$.

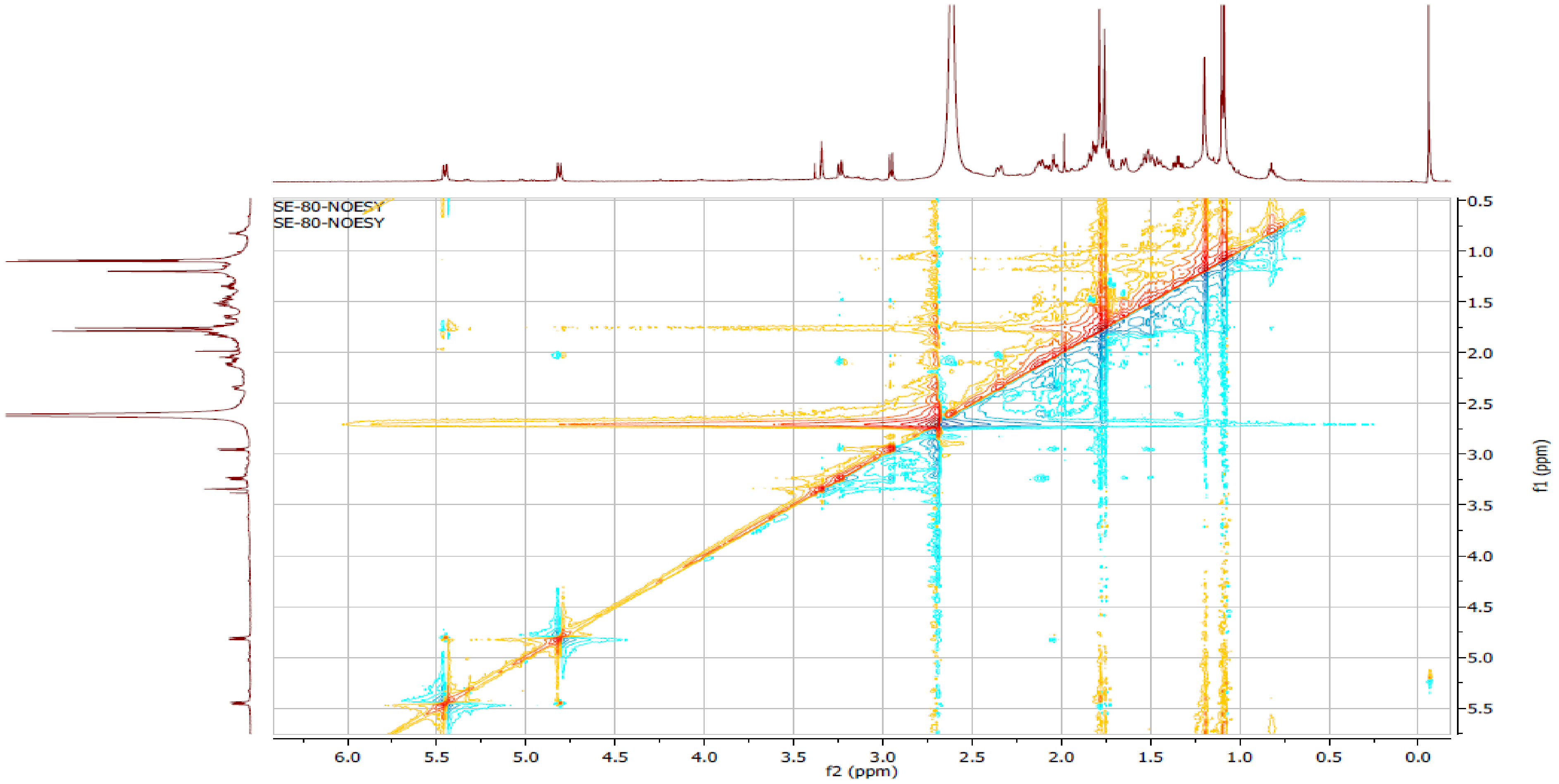

Figure S22. Optimized structure and relative free energy of conformers of **1** with Boltzmann population higher than 1%.

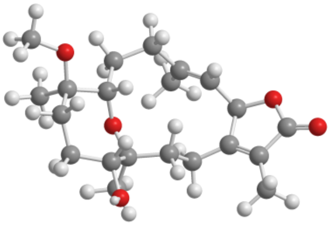

Compound 1a

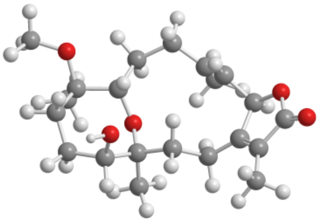

conformer 1b
Δ*G*=+2.8 kj/mol

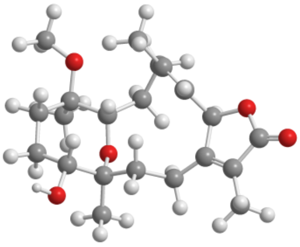

conformer 1c
Δ*G*=+3.1 kj/mol

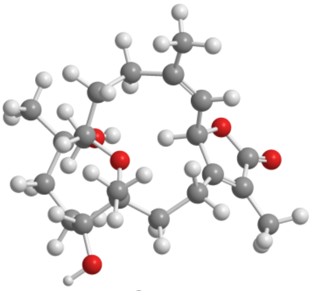

conformer 1d
Δ*G*=+3.2 kj/mol

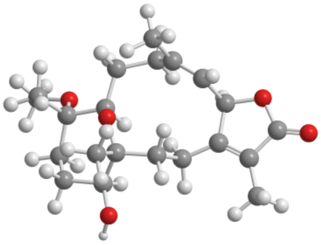

conformer 1e
Δ*G*=+4.2 kj/mol

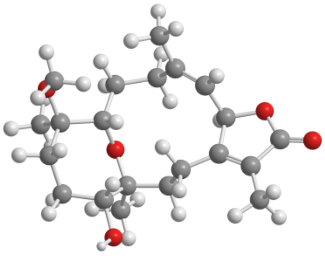

conformer 1f
Δ*G*=+4.3 kj/mol

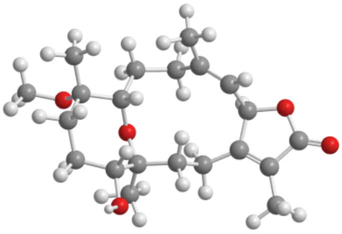

conformer 1g
Δ*G*=+5.3 kj/mol

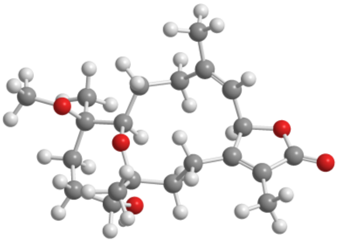

conformer 1h
Δ*G*=+7.0 kj/mol

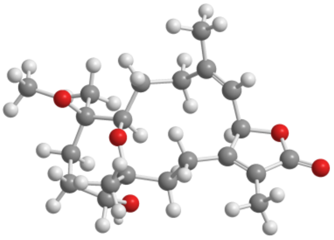

conformer 1i
Δ*G*=+7.4 kj/mol

Figure S23. Optimized structure and relative free energy of conformers of **2** with Boltzmann population higher than 1%.

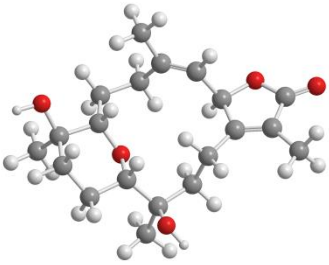

Compound 2a

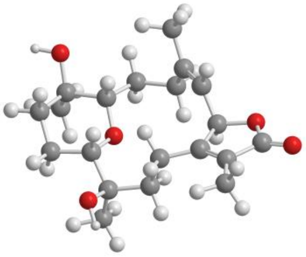

conformer 2b
Δ*G*=+0.2 kj/mol

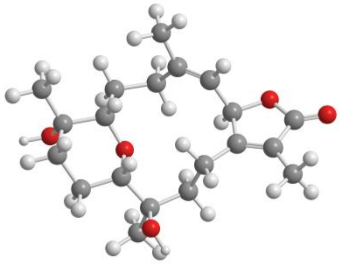

conformer 2c
Δ*G*=+1.5 kj/mol

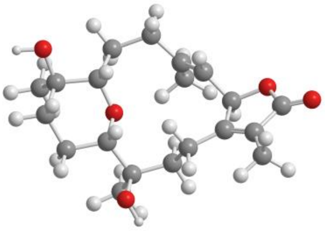

conformer 2d
Δ*G*=+4.4 kj/mol

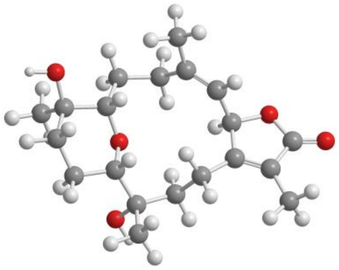

conformer 2e
Δ*G*=+6.6 kj/mol

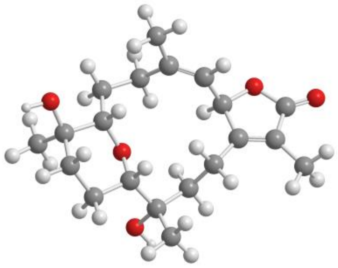

conformer 2f
Δ*G*=+6.8 kj/mol

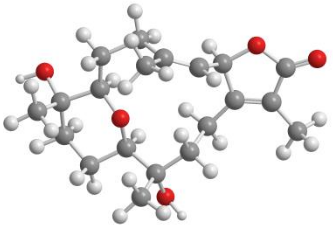

conformer 2g
Δ*G*=+7.4 kj/mol

Figure S24. Optimized structure and relative free energy of conformers of **3** with Boltzmann population higher than 1%.

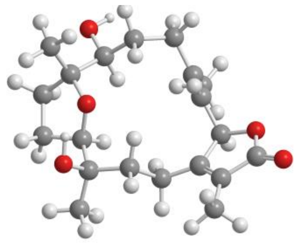

Compound 3a

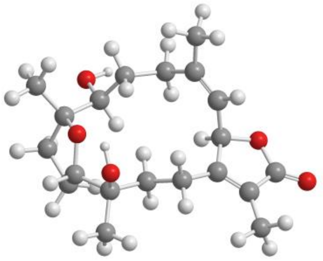

conformer 3b
Δ*G*=+0.9 kj/mol

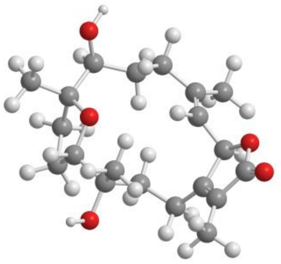

conformer 3c
Δ*G*=+4.0 kj/mol

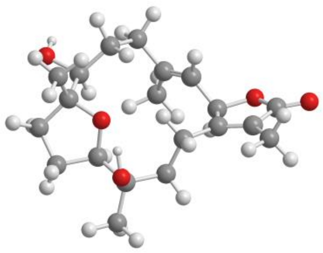

conformer 3d
Δ*G*=+5.5 kj/mol

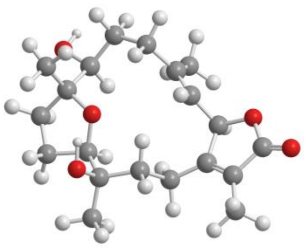

conformer 3e
Δ*G*=+5.9 kj/mol

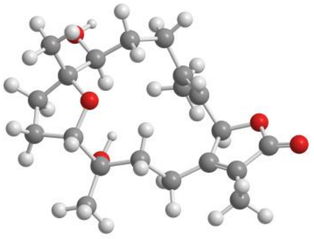

conformer 3f
Δ*G*=+6.0 kj/mol

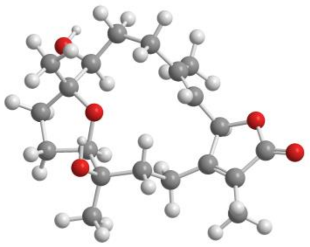

conformer 3g
Δ*G*=+6.0 kj/mol

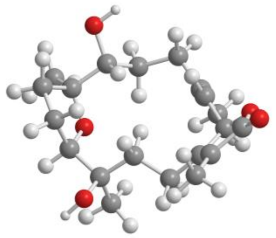

conformer 3h
Δ*G*=+6.7 kj/mol

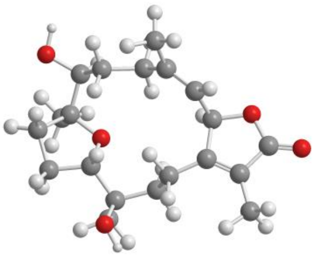

conformer 3i
Δ*G*=+6.9 kj/mol

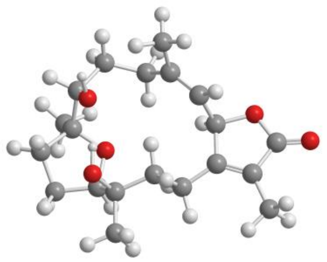

conformer 3j
Δ*G*=+8.6 kj/mol

Figure S25. Anti-proliferative A549 response curves with **1-8** based on MTT-reduction assay.

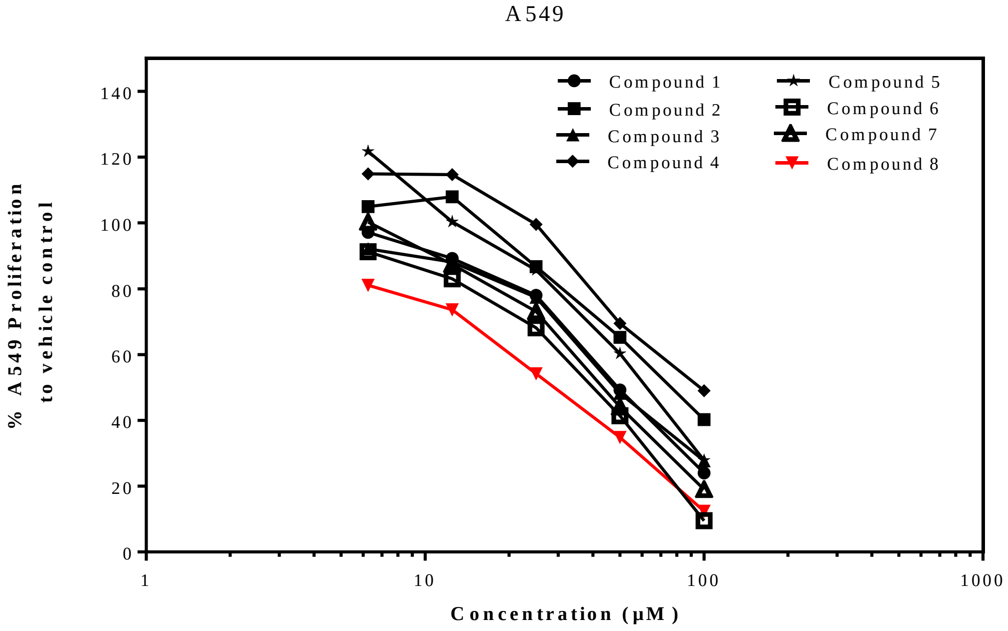

Figure S26. Anti-proliferative Caco-2 response curves with **1-8** based on MTT-reduction assay.

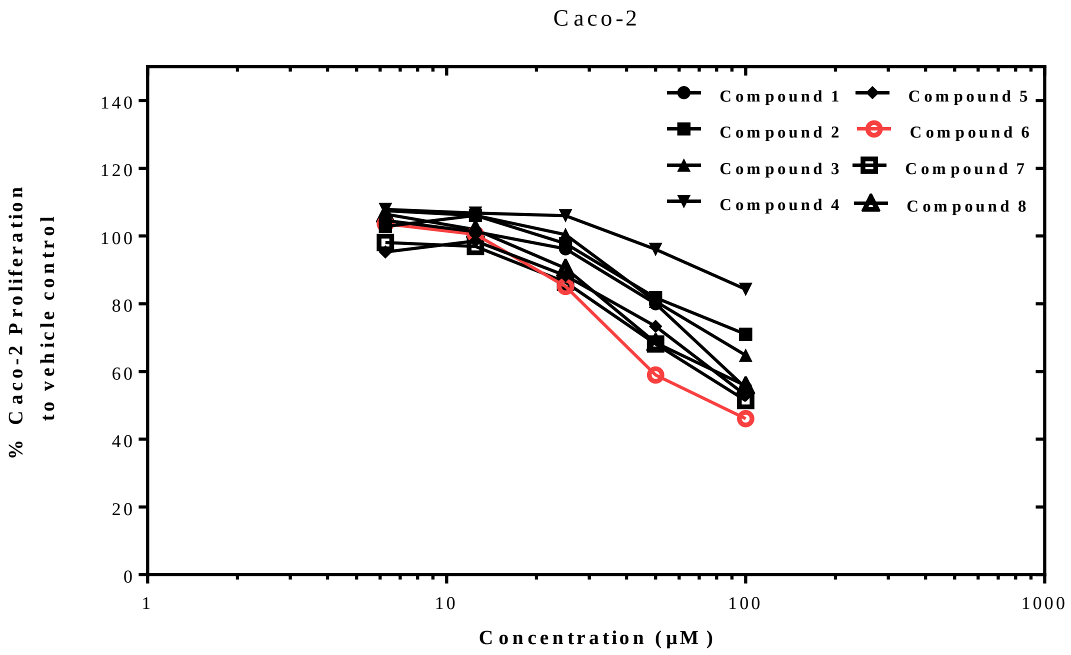

Figure S27. Anti-proliferative HepG2 response curves with **1-8** based on MTT-reduction assay.

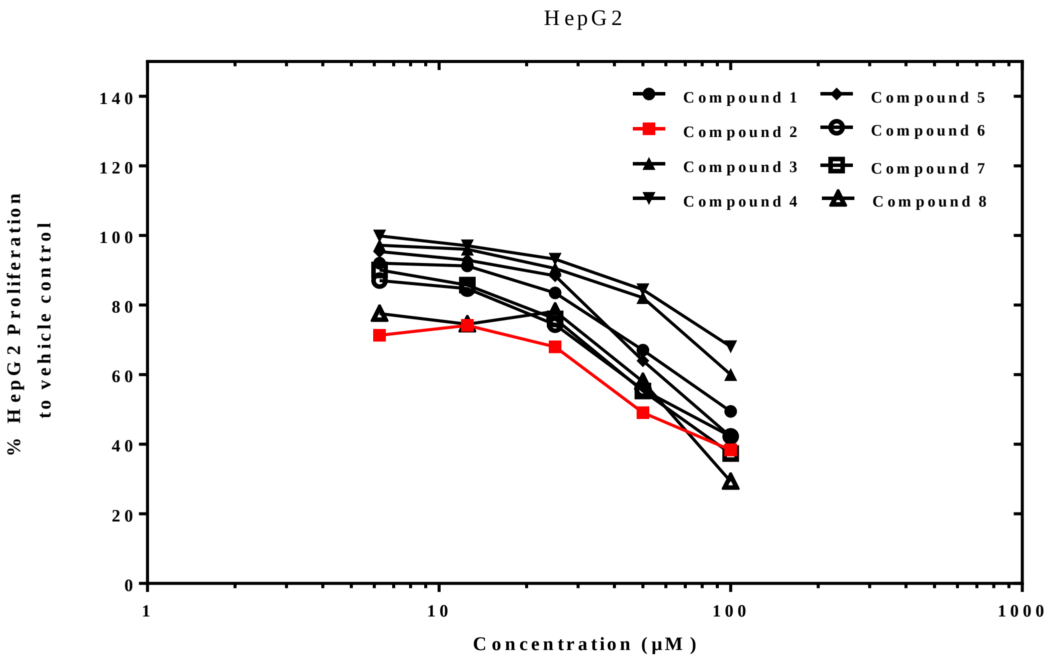

Figure S28. Calculated (i) Autodock and (ii) MM/GBSA binding energies of compounds with EGFR kinase domain relative to the experimental binding energies for the tested compounds against A549 cell line.

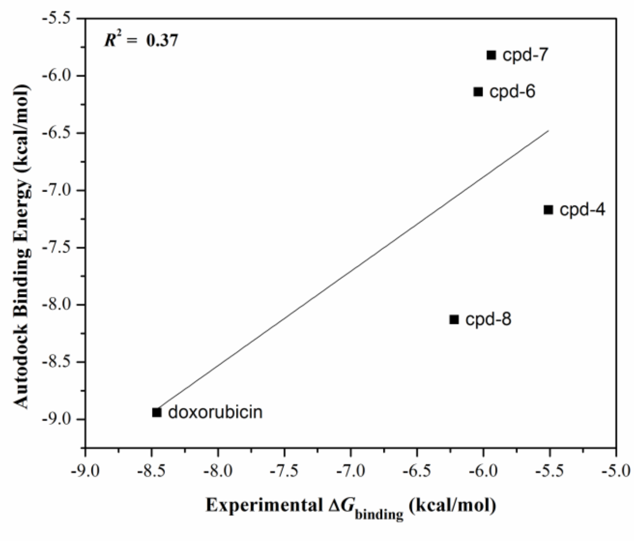

(i)

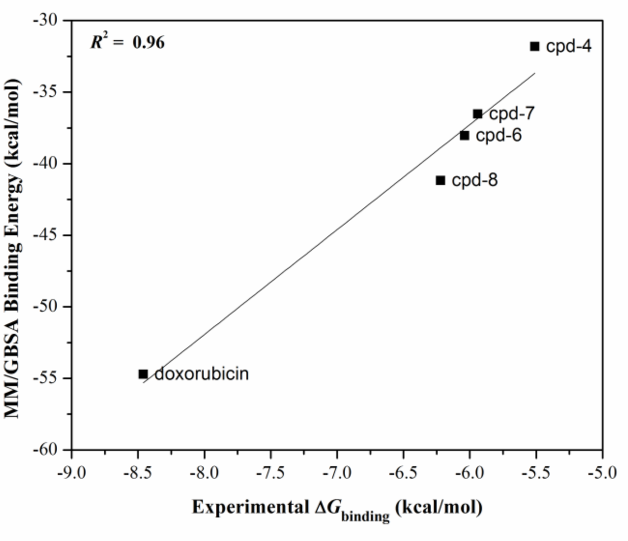

(ii)

Figure S29. Hydrogen bond distance between **8** and carboxylate oxygen atom of Asp_{776} inside EGFR active site over a 2.5 ns simulation time.

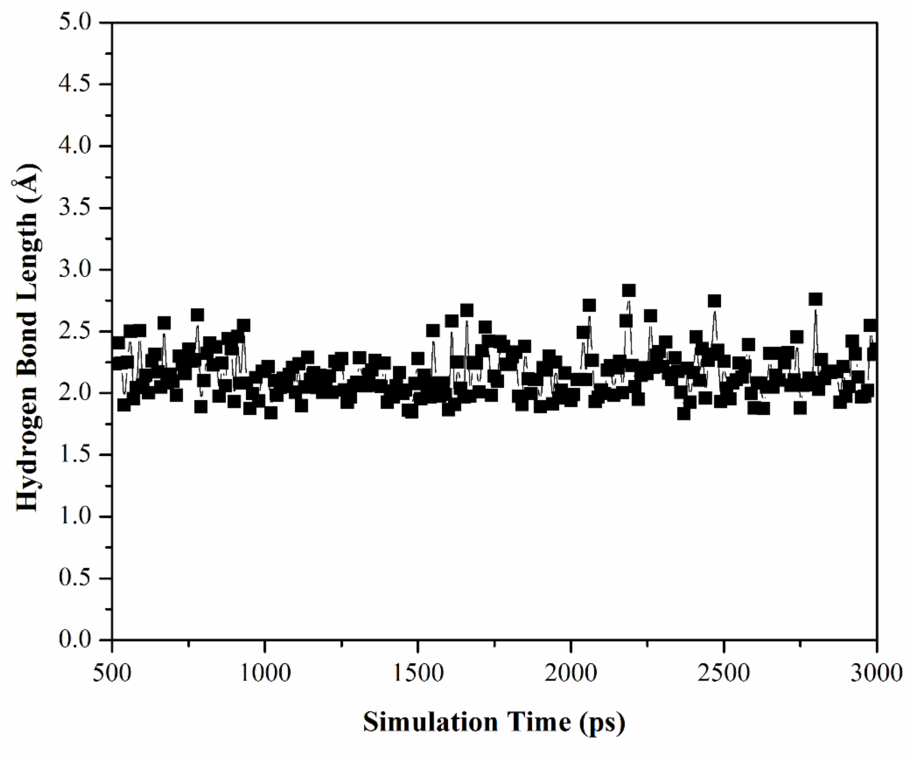

© 2017 by the authors; licensee MDPI, Basel, Switzerland. This article is an open access article distributed under the terms and conditions of the Creative Commons Attribution license (http://creativecommons.org/licenses/by/3.0/).
